# Novel Low‐Cytotoxic and Highly Efficient Type I Photoinitiators for Visible LED‐/Sunlight‐Induced Photopolymerization and High‐Precision 3D Printing

**DOI:** 10.1002/anie.202425598

**Published:** 2025-02-26

**Authors:** Tong Gao, Zheng Liu, Jiansong Yin, Ji Feng, Céline Dietlin, Fabrice Morlet‐Savary, Michael Schmitt, Tatiana Petithory, Laurent Pieuchot, Jing Zhang, Frédéric Dumur, Jacques Lalevée, Pu Xiao

**Affiliations:** ^1^ Université de Haute-Alsace, CNRS, IS2M UMR7361 F-68100 Mulhouse France; ^2^ Université de Strasbourg Strasbourg France; ^3^ Aix Marseille Univ, CNRS, ICR UMR 7273 F-13397 Marseille France; ^4^ Future Industries Institute University of South Australia Mawson Lakes SA 5095 Australia; ^5^ State Key Laboratory of High Performance Ceramics and Superfine Microstructure Shanghai Institute of Ceramics Chinese Academy of Sciences Shanghai 200050 P. R. China

**Keywords:** LED blue light, sunlight, low-cytotoxic, type I photoinitiators, free radical polymerization, glyoxylate and oxime ester, 3D printing, molecular modeling

## Abstract

The development of photoinitiators (PIs) combining high initiation ability, low‐toxicity, and availability for high‐precision 3D printing is a key challenge and an urgent problem to be solved nowadays in photopolymerization. In this study, carbazole chalcone glyoxylate oxime ester derivatives (denoted as Cs, C1−C5) containing both glyoxylate and oxime ester moieties with good light absorption properties in the visible range have been designed as type I PIs for the free radical photopolymerization (FRP) of trimethylolpropane triacrylate (TMPTA) and ethoxylated trimethylolpropane triacrylate (ETPTA) under 405 nm and 450 nm light‐emitting diodes (LEDs) as well as sunlight irradiation. The reaction properties and mechanism of Cs are firstly predicted by molecular modeling/molecular design, which anticipate that C5 could exhibit the best photoinitiation ability, this structure being more prone to decarboxylation. Subsequent experimental results clearly show that the photoinitiation ability of C5 outperforms that of the benchmark commercial PIs (methyl benzoylformate (MBF), diphenyl (2,4,6‐trimethylbenzoyl) phosphine oxide (TPO)), and phenylbis (2,4,6‐trimethylbenzoyl)‐phosphine oxide (BAPO) under the same conditions. Compared to TPO, the photoinitiation ability of C5 improved by 40 %, 132 %, and 47 % exposed to LED@405 nm, LED@450 nm, and sunlight. In addition, C5 is successfully applied to 3D printing for the manufacture of large‐scale and high‐resolution object. The photochemical mechanism of C5 is systematically and comprehensively analyzed using a combination of steady state photolysis, decarboxylation reaction, fluorescence experiments, and electron spin resonance‐spin trapping (ESR‐ST) technology. It is found that both glyoxylate and oxime ester in C5 are highly active and capable of undergoing decarboxylation reactions to produce CO_2_ and free radicals, which is consistent with the results predicted by molecular modeling. Furthermore, the low‐toxicity of C5 is evidenced by cytotoxicity assays. The comprehensive molecular modeling and experimental approach adopted in this research has led to the development of novel PIs that are highly efficient and low‐toxic, and can be used for high‐precision 3D printing, which offers broad application prospects in the fields of environmental sustainability, visible light curing, and biomedical science.

## Graphical abstract

This study develops new photoinitiators (PIs) for photopolymerization, combining high efficiency, low toxicity, and compatibility with 3D printing. The C5 compound, designed via molecular modeling, outperforms commercial PIs, improving initiation by 40–132 % under LED and sunlight. Its efficiency, photochemical mechanism, and low toxicity are experimentally confirmed.

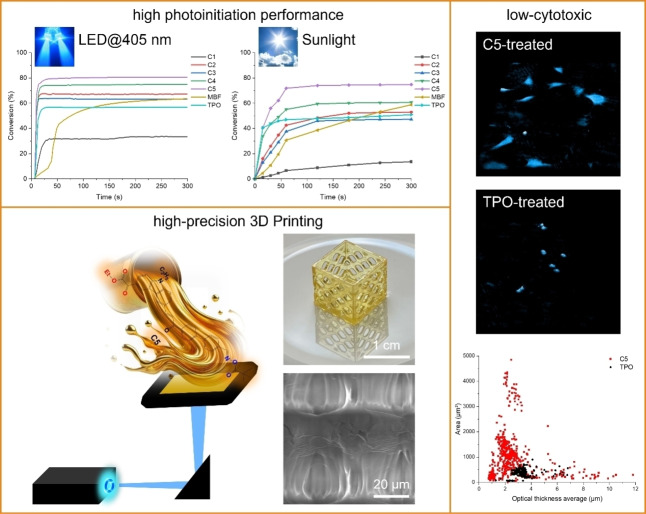

## Introduction

Photopolymerization is a highly efficient technology that uses light radiation to initiate the polymerization reaction of monomers/oligomers in the presence of photoinitiators (PIs).[^1^, ^2^] It has been widely used in coatings and inks,[^3^, ^4^] adhesives,^[5]^ 3D printing,[^6^, ^7^, ^8^] biomedical engineering,[^9^, ^10^, ^11^] metamaterials,[^12^, ^13^] dental materials,^[14]^ photoresists,^[15]^ and relative fields[^16^, ^17^] owing to its advantages of fast reaction speed, spatiotemporal controllability, and energy saving.[^18^, ^19^, ^20^] Light sources and PIs are essential parts of photopolymerization systems. The light source can be ultraviolet (UV) lamps, artificial visible light‐emitting diodes (LEDs), or natural sunlight.^[21]^ Compared to traditional UV light sources, LED light sources are more wavelength stable and suitable for precise control of polymerization reactions. Moreover, LEDs are much more environmentally friendly, low cost and low energy consumption, making them a significantly more sustainable option.[^22^, ^23^] Full‐spectrum sunlight, as a natural light source, without financial expense and generally readily available outdoors. Hence its use can meet the demand for low cost, environmentally friendly applications, and is especially suitable for large area outdoor light curing applications.[^24^, ^25^] Different light sources have different wavelengths and energy distributions, so it is very critical to choose a suitable light source to match the absorption spectra of the PIs. The sun as an acceptable blackbody irradiator has a very broad spectral range, which is well documented, with the maximal energy density of direct and diffuse irradiation ranging from around 800 W/m^2^ to 450 W/m^2^ from summer to winter for the approximate local position where the experiments were conducted.^[26]^

Photoinitiators are very critical components in photopolymerization, responsible for absorbing light energy and generating reactive species (e.g., free radicals or cations) to initiate the polymerization reaction.^[27]^ According to their reaction mechanisms, PIs are mainly classified into Type I PIs which directly undergo α‐cleavage to generate free radicals by absorbing light energy, and Type II PIs reacting with co‐initiators to generate free radicals by hydrogen abstraction after light absorption.[^28^, ^29^, ^30^] However, the application of Type II PIs in complex systems is limited by the need of co‐initiators and the issue of photosensitivity in addition to oxygen sensitivity.^[31]^ On the contrary, Type I PIs generate free radicals through a single photolysis reaction, avoiding the use of co‐initiators. For example, acylgermylenes have gained significant attention as a promising class of photoinitiators due to their outstanding absorption properties and high efficiency in generating reactive radicals.^[32]^ Therefore, type I PIs are particularly suitable for modern visible light and green photopolymerization processes as they benefit from the advantages of fast reaction speeds and suitability for use in complex environments (e.g., visible light and airborne applications).

Oxime esters, with easy synthesis, high thermal stability and photoactivity, have been widely studied as typical Type I PIs and are in widespread use in free radical photopolymerization reactions.[^33^, ^34^, ^35^] However, the commercial oxime PIs Irgacure®OXE‐01 and Irgacure®OXE‐02 have absorption bands located in the ultraviolet range (<360 nm), which lead to lower initiation ability in free radical photopolymerization reactions under near‐ultraviolet or visible LED irradiation.[^36^, ^37^, ^38^] Although different chromophores (e.g. coumarin,^[39]^ phenothiazine,^[40]^ and carbazole^[41]^) can be used for the modification of the oxime esters, most of the different oxime ester‐based PIs prepared can be used for photopolymerization under 405 nm visible LED radiation.^[42]^ A few oxime esters can initiate photopolymerization under longer wavelength visible LED irradiation (e.g., 425 nm and 455 nm visible LEDs).[^43^, ^44^] However, only a limited number are effective under sunlight.^[45]^ In addition, the cytotoxicity of PIs has a critical impact on their potential applications in many fields: inks, coatings, medical devices, dental materials, clinical therapy, and biological tissue engineering.[^46^, ^47^, ^48^] Thus, it is very valuable and imperative to develop new type I PIs with high initiation performance and low‐toxicity under long‐wavelength visible LEDs and sunlight irradiation, and even capable of being applied to 3D printing.

**Scheme 1:**
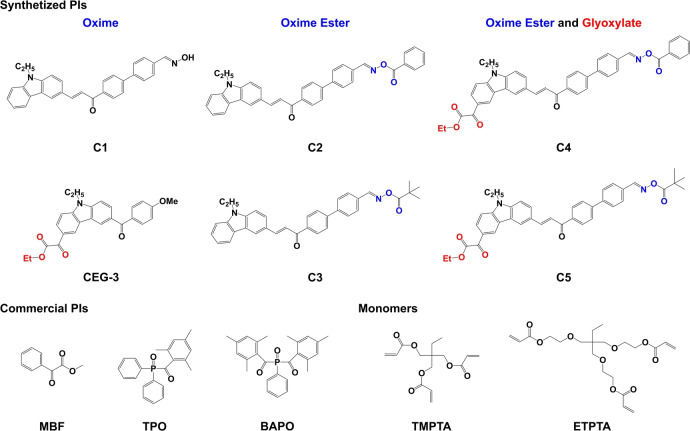
Chemical structures of the synthesized carbazole chalcone glyoxylates and oxime esters derivatives (Cs), commercial PIs (MBF, TPO, and BAPO), and monomers (TMPTA and ETPTA) used in this study, as well as the structure of CEG‐3 from the previous study.^[30]^

In this study, we successfully designed and developed a series of carbazole chalcone glyoxylate oxime compounds (denoted as Cs compounds, C1−C5) (See Scheme 1) as efficient Type I PIs, which have never been reported in the literature before. In our previous work, we reported carbazole‐based ethyl glyoxylate derivatives (CEG‐3) as highly efficient PIs for free radical photopolymerization (FRP) reactions exposed to LED@405 nm.^[30]^ Inspired by this, with the introduction of glyoxylate moiety into the compounds containing oxime ester moiety to enhance the light absorption properties in this study, the prepared compounds contain both oxime ester and glyoxylate moieties exhibited high activity. The reactivity and mechanism of C5 were first predicted by molecular modeling calculations in computational chemistry. Then, with methyl benzoylformate (MBF), diphenyl (2,4,6‐trimethylbenzoyl) phosphine oxide (TPO), and phenylbis (2,4,6‐trimethylbenzoyl)‐phosphine oxide (BAPO) commercial PIs as benchmarks, the photoinitiation properties of C5 were studied using real‐time Fourier‐transform infrared spectroscopy (RT‐FTIR) under blue light and sunlight irradiation for the FRP of trimethylolpropane triacrylate (TMPTA) and ethoxylated trimethylolpropane triacrylate (ETPTA) with more flexible ethylene glycol units.^[49]^ Next, 3D printing and direct laser write (DLW) experiments were conducted. To thoroughly investigate the photochemical mechanism of C5, a combination of techniques was employed, including UV/Visible spectroscopy, decarboxylation analysis, steady state photolysis, fluorescence experiments, electron spin resonance‐spin trapping (ESR‐ST), and molecular modeling. Finally, C5 was studied for its cytotoxicity. Based on the above findings, it can be concluded that C5 not only presented the high photoinitiation ability under blue light and sunlight, but was also suitable for the high precision 3D printing as well as low‐toxic. These comprehensive properties in one package made C5 an exceptional candidate for photopolymerization with good development potential and broad application prospects.

## Results and Discussion

### Synthesis of Cs

**Scheme 2:**
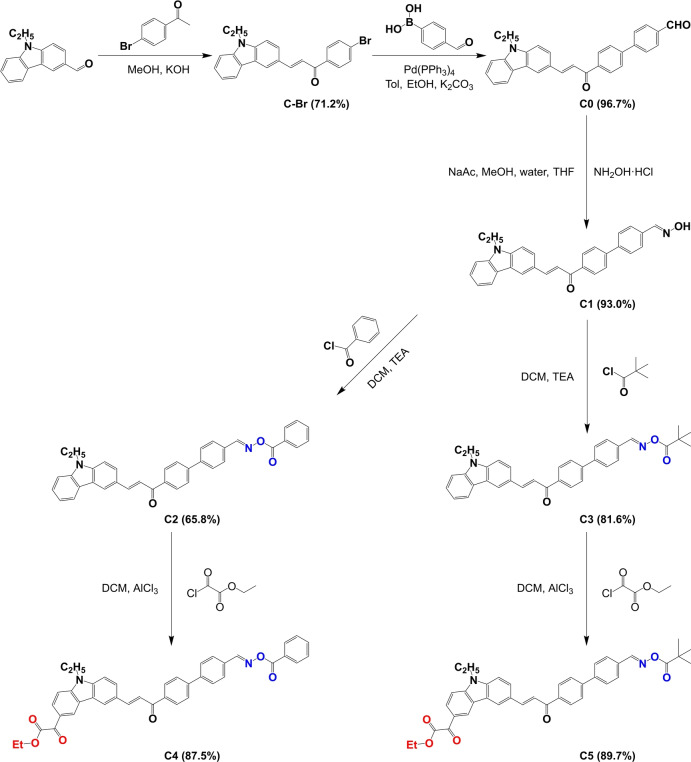
Synthetic procedure for Cs and their preparation yields.

Scheme 2 briefly summarizes the synthetic routes for the different target compounds. Supporting Information provides details of the synthesis steps and their characterization. Firstly, C−Br was synthesized by a Claisen‐Schmidt condensation using 9‐ethyl‐9*H*‐carbazole‐3‐carbaldehyde with 4′‐bromoacetophenone under alkaline (KOH) conditions. Afterwards, C0 was synthesized by a Suzuki–Miyaura Cross‐Coupling reaction using Pd(PPh_3_)_4_ as the metal catalyst, C−Br and 4‐formylphenylboronic acid for the cross‐coupling reaction and K_2_CO_3_ as the base. Then, C0 was reacted with NH_2_OH⋅HCl to produce C1 containing an oxime moiety. C1 was then converted as oxime esters using benzoyl chloride or pivaloyl chloride as the acid chlorides, enabling to produce C2 and C3 containing the oxime ester moiety. Subsequently, by a Friedel‐Craft reaction using ethyl chlorooxoacetate, C2 and C3 were converted as C4 and C5 containing both oxime ester and glyoxylate moieties, respectively. Through this step‐by‐step reaction strategy, compounds containing only oxime moiety, only oxime ester moiety and both oxime ester and glyoxylate moieties were successfully realized, ensuring the functional diversity of the products.

### Molecular Design/Molecular Modeling

**Scheme 3:**
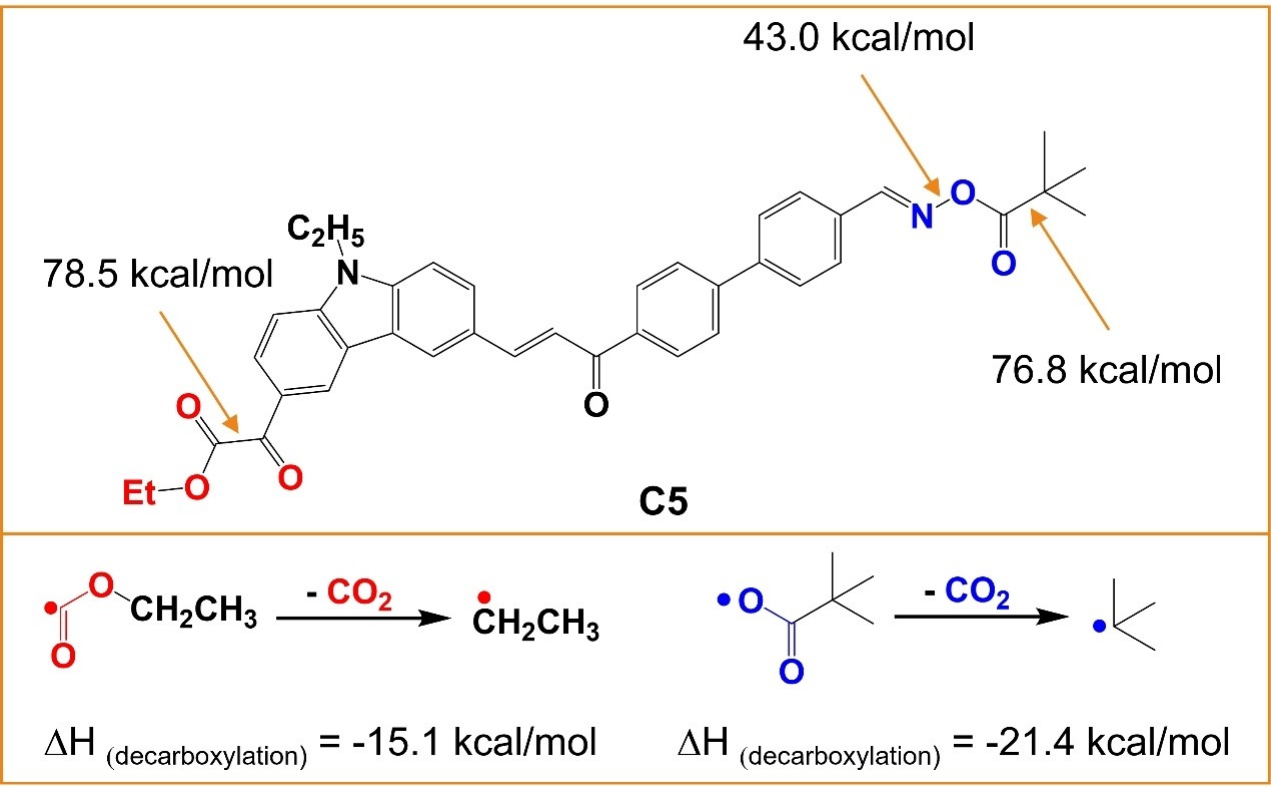
BDE and ΔH_decarboxylation_ of the glyoxylate and oxime ester chromophores. Optimization was carried out at the B3LYP/6‐31G* level.

The photoinitiation properties and the proposed mechanisms of the Cs compounds were predicted by molecular modeling of theoretical calculations. The predicted results (See Table 1) show that C1 containing neither oxime ester nor glyoxylate moiety did not undergo a decarboxylation reaction, and the Bond Dissociation Energies (BDE) of N−O bond was higher than that of other Cs compounds. For C2 (oxime ester attached to phenyl group) and C3 (oxime ester attached to *tert*‐butyl group) containing only oxime ester moiety, the enthalpy of decarboxylation (ΔH_decarboxylation_) of C2 was much larger than that of C3. It was due to the fact that the phenyl group of C2 may enhance the stability of the oxime ester and reduce the probability of the occurrence of decarboxylation. For C4 (oxime ester linked to phenyl group) and C5 (oxime ester linked to *tert*‐butyl group) containing both oxime ester and glyoxylate moieties, the ΔH_decarboxylation_ of the oxime ester moiety in C4 was the same as that of in C2, but lower than that of in C5. It was because the *tert*‐butyl group attached to the oxime ester moiety in C5 was less stable for delocalization compared to the phenyl group, making the *tert*‐butyl radical more reactive and the oxime ester moiety more susceptible to decarboxylation.^[50]^ Remarkably, C5 had lower ΔH_decarboxylation_ for both oxime ester and glyoxylate moieties (See Scheme 3). Based on these theoretical calculations, it was boldly predicted that C5 had the best photoinitiation ability and its photochemical mechanism must include the decarboxylation of oxime ester and glyoxylate moieties.

**Table 1:** BDE of N−O bond in the oxime ester moiety and C−C bond in the glyoxylate moiety, ΔH_decarboxylation_ of ⋅OC(=O)R in the oxime ester moiety and ⋅C(=O)OEt in the glyoxylate moiety.

Cs	N−O BDE (kcal/mol)	C−C BDE (kcal/mol)	ΔH_decarboxylation oxime ester_ (kcal/mol)	ΔH_decarboxylation glyoxylate_ (kcal/mol)
C1	65.3	n.d.	n.d.	n.d.
C2	42.9	n.d.	−0.01	n.d.
C3	43.0	n.d.	−21.4	n.d.
C4	43.0	78.5	−0.01	−15.1
C5	43.0	78.5	−21.4	−15.1

n.d.: not determined.

**Figure 1:**
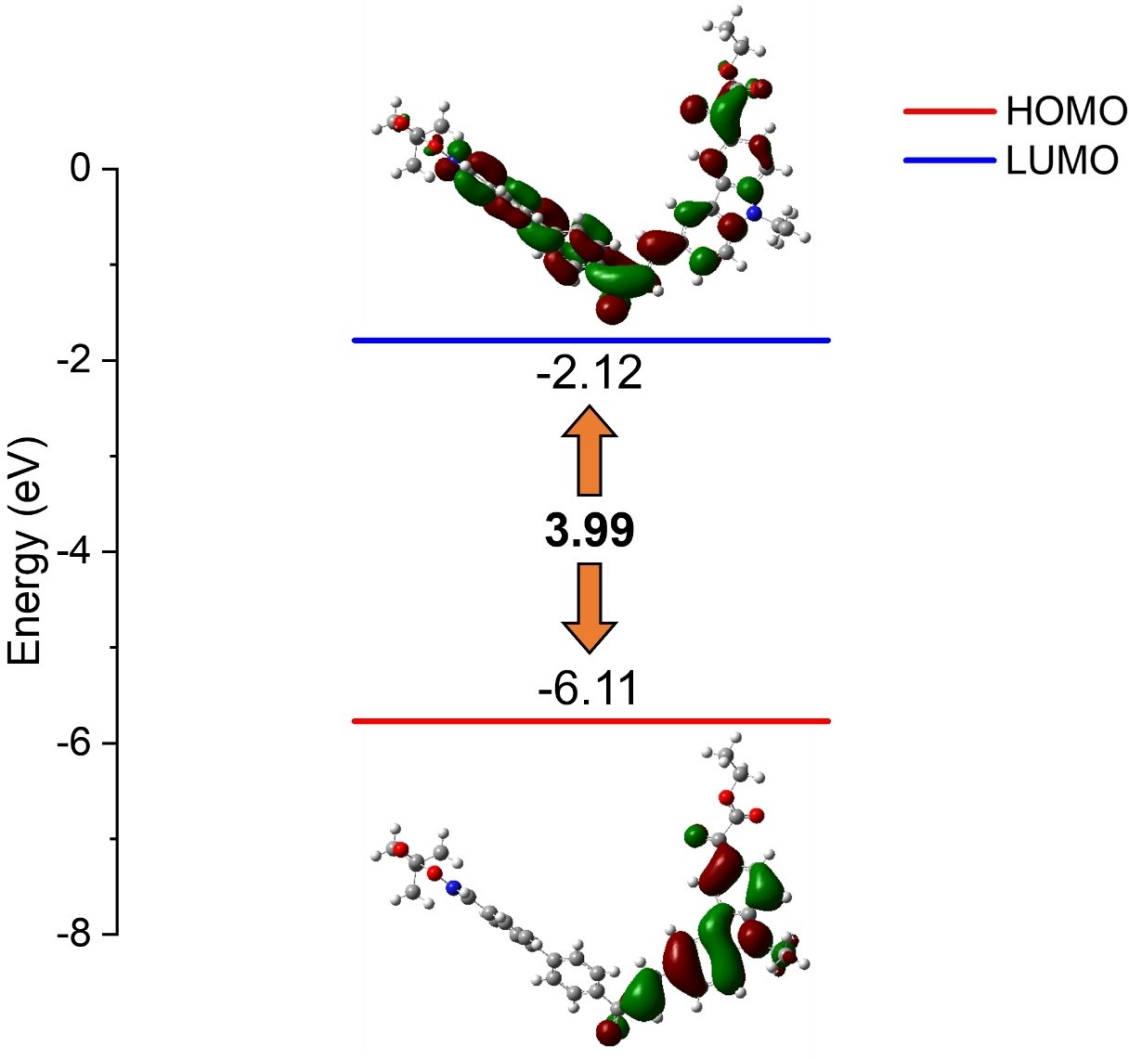
The HOMO, LUMO, and energy gap of C5. Optimization was carried out at the B3LYP/6‐31G* level and orbitals were optimized at the MPW1PW91/6‐31G* level of theory at a single point (isovalue=0.02).

By analyzing the frontier molecular orbitals of Cs, with the highest occupied molecular orbital (HOMO) and the lowest unoccupied molecular orbital (LUMO), the energy gaps of all the Cs (See Table S1) was lower than that of OXE02 (4.20 eV), suggesting that the maximum absorption wavelengths of the Cs might experience a redshift.^[51]^ Figure 1 and Figure S1 show not only the typical π–π* transition, but also the intramolecular charge transfer (ICT) transition. The HOMO electron clouds were mainly concentrated on carbazole and vinyl ketone groups for all Cs. The LUMO electron clouds were mainly concentrated on the chalcone phenyl and to a lesser extent on the carbazole moiety for C1, C2 and C3. Compared to this, the LUMO electron clouds of C4 and C5 was not only mainly concentrated on chalcone phenyl groups, but also a large number of electron clouds on carbazole moiety. The carbazole moiety acted as an electron donor, whereas the chalcone or oxime ester or the glyoxylate moiety can act as an electron acceptor. Therefore, the push‐pull effect led to the ICT transition bands characterized by π–π* interactions overlaying the n‐π* transition bands. In the UV/Visible absorption spectra of Cs, the absorption bands of C4 and C5 may be enhanced in intensity, suggesting that the transition occurred with high extinction coefficients.

### Photophysical Properties

**Figure 2:**
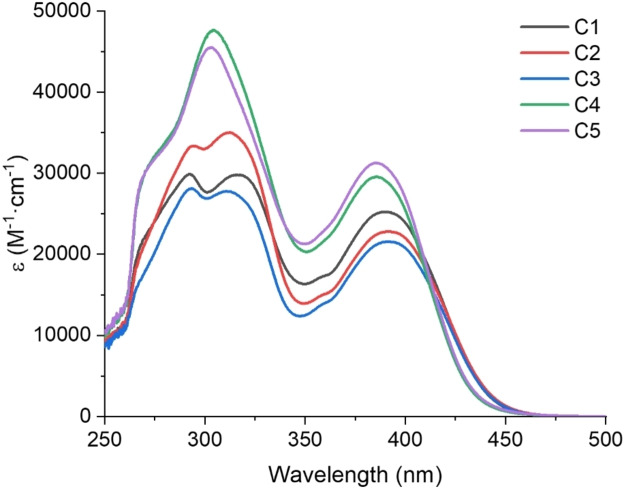
UV/Visible absorption spectra of Cs in ACN (concentration=5×10^−5^ M).

Figure 2 describes the UV/Visible absorption spectra of the PIs in acetonitrile (ACN). Table 2 presents the specific photophysical properties for these compounds, i.e., maximum absorption wavelength (λ_max_), and molar extinction coefficient at the maximum absorption wavelength (ϵ_max_), 405 nm absorption wavelength (ϵ_405_), and 450 nm absorption wavelength (ϵ_450_). The λ_max_ of these PIs were longer than that of MBF (λ_max_=266 nm),^[30]^ which were attributed to the presence of large carbazole and chalcone conjugate groups in these compounds. This result verified the redshift that occurred in the theoretical calculations of molecular orbitals. The maximum absorption wavelengths of these PIs were close to 405 nm, ranging from 385 nm to 395 nm, and the shape of the absorption peaks in the UV/Visible region showed significant similarity (See Figure 2). The ϵ_max_ of C4 and C5 were higher than that of C1, C2 and C3, which was in line with the theoretical calculations. The ϵ_405_ of these PIs were higher than 19 000 M^−1^ cm^−1^, and especially the ϵ_405_ of C5 reached the maximum, up to 23 200 M^−1^ cm^−1^. Abovementioned results indicated that these PIs had good absorption properties at 405 nm and for near UV LEDs (365, 385 or 395 nm), and the conjugation effect would cause the maximum absorption wavelength to be redshifted. Moreover, the absorption bands of these PIs extended beyond 450 nm, with absorption at 450 nm as well. Therefore, LED@405 nm and LED@450 nm were used as light sources in the subsequent photopolymerization study.

**Table 2:** Photophysical properties of Cs in ACN (concentration=5×10^−5^ M).

Cs	λ_max_ (nm)	ϵ_max_ (M^−1^ cm^−1^)	ϵ_405_ (M^−1^ cm^−1^)	ϵ_450_ (M^−1^ cm^−1^)
C1	391	25 200	21 900	1 100
C2	391	22 800	20 500	1 300
C3	392	21 600	19 300	1 200
C4	386	29 600	22 000	600
C5	386	31 300	23 200	800

### Violet and Blue Light‐Induced Photopolymerization

**Figure 3:**
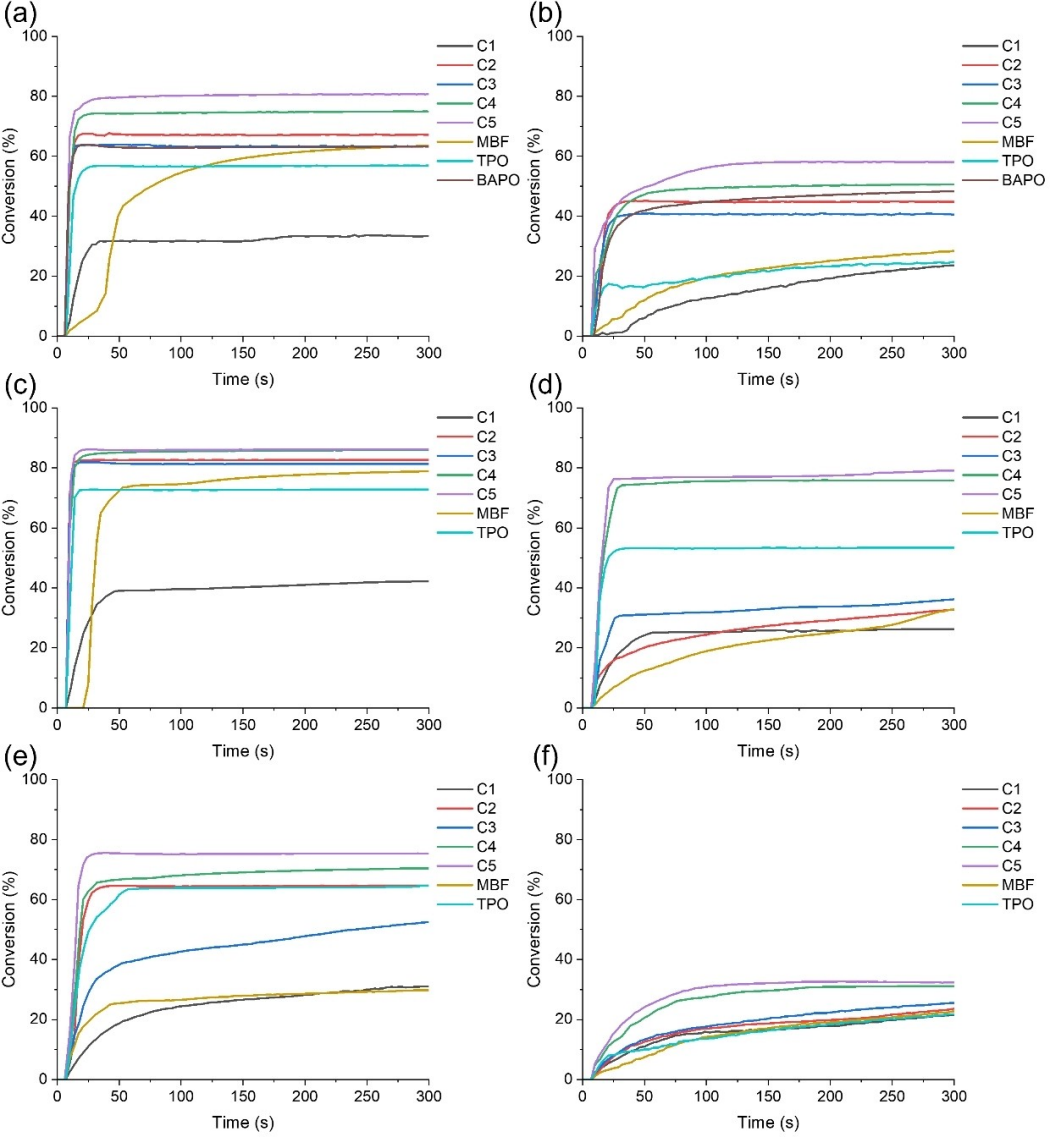
Photopolymerization kinetics of TMPTA with PIs (1×10^−5^ mol g^−1^ TMPTA) in laminate (thickness ∼25 μm) exposed to (a) LED@405 nm and (b) LED@450 nm; photopolymerization kinetics of ETPTA with PIs (1×10^−5^ mol g^−1^ ETPTA) in laminate (thickness ∼25 μm) exposed to (c) LED@405 nm and (d) LED@450 nm; photopolymerization kinetics of ETPTA with PIs (1×10^−6^ mol g^−1^ ETPTA) in laminate (thickness ∼25 μm) exposed to (e) LED@405 nm and (f) LED@450 nm; TMPTA and ETPTA as monomers. Exposure starts at t=10 s.

The photoinitiation ability of Cs for TMPTA and ETPTA monomers was studied using LED@405 nm (110 mW cm^−2^) and LED@450 nm (50 mW cm^−2^) as light sources, with MBF, TPO, and BAPO commercial PIs serving as controls. Figure 3 shows the kinetics of TMPTA and ETPTA photopolymerization with various concentrations of PIs exposed to LED@405 nm and LED@450 nm. Table 3 summarizes the details of the final acrylate function conversions (Conv) in different Cs/TMPTA and Cs/ETPTA.

**Table 3:** The Conv of TMPTA and ETPTA with different concentrations of PIs exposed to LED@405 nm and LED@450 nm.

PIs	PIs 1×10^−5^ mol g^−1^ TMPTA		PIs 1×10^−5^ mol g^−1^ ETPTA		PIs 1×10^−6^ mol g^−1^ ETPTA	
	LED@405 nm	LED@450 nm	LED@405 nm	LED@450 nm	LED@405 nm	LED@450 nm
C1	33	24	42	31	27	22
C2	67	45	83	65	33	23
C3	63	41	81	46	36	26
C4	75	51	86	70	76	31
C5	80	58	86	75	79	32
MBF	63	28	79	30	33	23
TPO	57	25	73	65	53	22
BAPO	63	48	n.d.	n.d.	n.d.	n.d.

n.d.: not determined.

**Figure 4:**
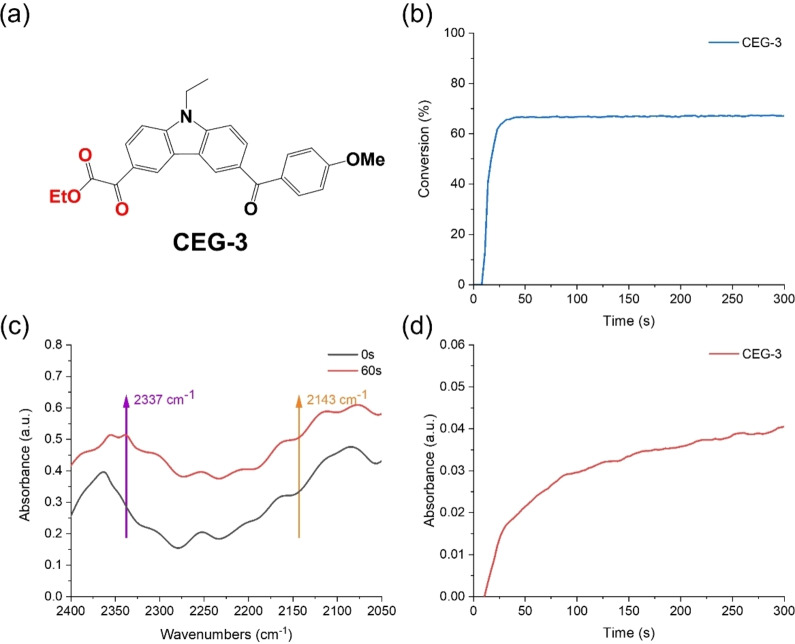
(a) Chemical structures of CEG‐3;^[30]^ (b) photopolymerization kinetics of TMPTA in laminate (thickness ∼25 μm) exposed to LED@405 nm with CEG‐3 (1.25×10^−5^ mol g^−1^ TMPTA); (c) Infrared spectra of CEG‐3 in TMPTA at t=0 s and 60 s; (d) The curves of absorption intensity of CO_2_ derived from CEG‐3.^[30]^

The Conv of the Cs/TMPTA were larger than those of MBF/TMPTA and TPO/TMPTA when exposed to LED@405 nm and LED@450 nm irradiation, except for C1/TMPTA. The Conv of C5/TMPTA can reach 80 %, which were much higher than those of not only MBF/TMPTA and TPO/TMPTA, but also other Cs/TMPTA under LED@405 nm irradiation (See Figure 3a). This was possible not only due to the excellent light absorption property of C5 at 405 nm wavelength, but also due to its good solubility of C5, allowing it to be completely dissolved in TMPTA. Only C5 can be perfectly dissolved in TMPTA when the concentration of PIs was 1×10^−5^ mol g^−1^ TMPTA (See Figure S2 and Table S2). In addition, it was also attributed to the fact that C5 consisted of both glyoxylate and oxime ester moieties as opposed to C2 and C3, which contained only one moiety of oxime ester. However, the Conv of C4/TMPTA was lower than that of C5/TMPTA, which was affected not only by the light absorption property, but also by the solubility, with C4 not fully dissolved in TMPTA. C1/TMPTA exhibited the lowest Conv, which was probably attributed to the fact that neither glyoxylate nor oxime ester moieties were available in C1. Additionally, CEG‐3, which only contained glyoxylate in a previous study,^[30]^ was analyzed in comparison with C5 in this study. It was found that the Conv of CEG‐3/TMPTA was lower than that of C5/TMPTA, indicating the superior photoinitiation ability of C5 (See Figures 4a and 4b).^[30]^ The photoinitiation abilities of these PIs exposed to LED@450 nm (See Figure 3b) were remarkably similar to that upon the exposure to LED@405 nm. Moreover, C5 also performed well with good photoinitiation ability under LED@450 nm irradiation, and its Conv can be up to 58 %, which was higher than the Conv of MBF/TMPTA and TPO/TMPTA. In addition, BAPO was also used as a benchmark system. Interestingly, the Conversion of C5/TMPTA was also superior to that of BAPO/TMPTA in the photopolymerization experiments exposed to LED@405 nm and LED@450 nm (See Figures 3a and 3b). The above results suggested that C5 outperformed TPO, MBF, and BAPO commercial PIs in the photopolymerization experiments under LED@405 nm and LED@450 nm irradiation.

To minimize the effect of the solubility of the PIs in the monomers on the initiation ability, further investigation was conducted by introducing ETPTA containing more flexible chain segments (ethoxylated chains) as a monomer and decreasing the concentrations of the PIs. For PIs concentrations of 1×10^−5^ mol g^−1^, C2, C4, and C5 can be perfectly dissolved in ETPTA. When the PIs concentrations were lowered to 1×10^−6^ mol g^−1^, all PIs can be completely dissolved in ETPTA (See Figure S2). When the PIs concentrations were 1×10^−5^ mol g^−1^, the Conv of Cs/ ETPTA was higher than that of MBF/ETPTA and TPO/ETPTA exposed to LED@405 nm, except for C1/ETPTA (See Figure 3c). And the Conv of C5/ETPTA and C4/ETPTA were the same, both up to 86 %. C5/ETPTA and C4/ETPTA also demonstrated high polymerization rates under LED@450 nm irradiation (See Figure 3d). When the concentration of PIs was reduced to 1×10^−6^ mol g^−1^, the Conv of all Cs/ETPTA under LED@405 nm irradiation was lower than that of Cs/ETPTA with the photoinitiator concentration of 1×10^−5^ mol g^−1^. However, the Conv of C5/ETPTA and C4/ETPTA were still as high as 79 % and 76 %, respectively (See Figure 3e). Because of the low concentration of PIs, the Conv of Cs/ETPTA also decreased under the LED@450 nm irradiation (See Figure 3f). The above results indicated that the photoinitiation properties of the compounds containing glyoxylate and oxime ester moieties were superior to those of the compounds containing only single oxime ester moiety.

In summary, it can be concluded that among these PIs, C5 showed the best photoinitiation ability. Significantly, the PIs had excellent light absorption properties and good solubility as well as the presence of both glyoxylate and oxime ester moieties would enhance their initiation ability.

### Sunlight‐Induced Photopolymerization

**Figure 5:**
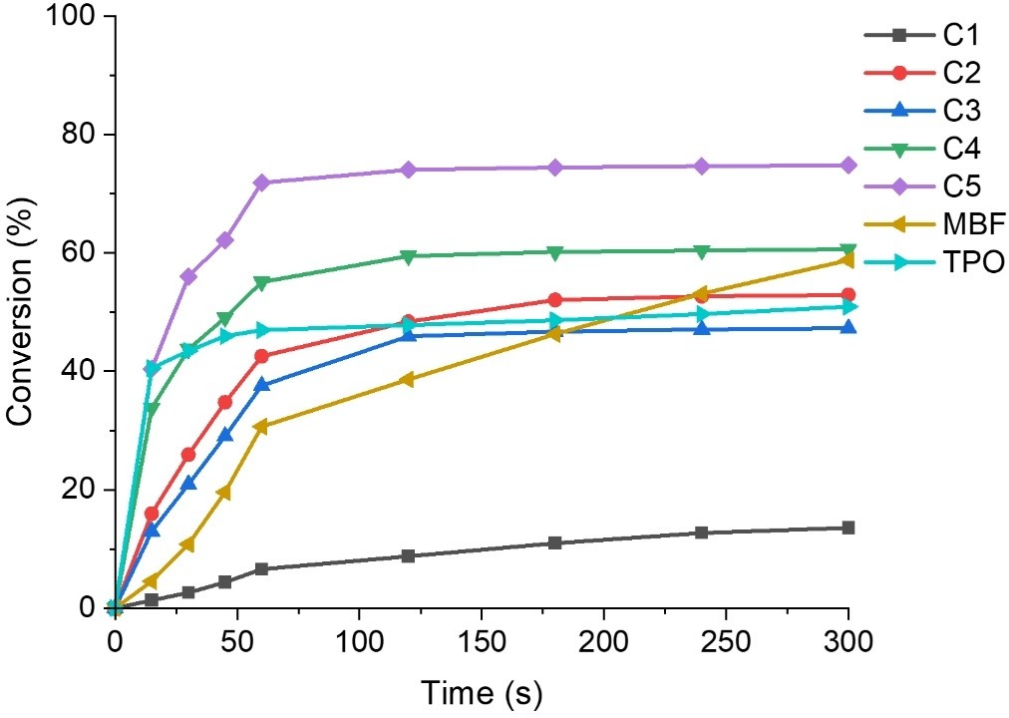
Photopolymerization kinetics (acrylate function conversion) for TMPTA with PIs (1×10^−5^ mol g^−1^ TMPTA) in laminate (thickness ∼25 μm) after different times of irradiation by sunlight.

The photoinitiation abilities of the PIs exposed to sunlight were studied using the same formulations under the LED irradiation. Figure 5 and Table 4 presented the results of Cs/TMPTA photopolymerization kinetics under sunlight. The experimental site was located at 47°43′47′′ N and 7°18′35′′ E with sunny weather. The specific experimental conditions are shown in Figure S3. Except for C1, the other Cs behaved well under sunlight irradiation, and the Conv of C5/TMPTA could reach 70 % and 73 % after 60 s and 300 s. These PIs showed remarkable similarity in the trend of initiation behavior when exposed to sunlight with that exposed to LED artificial light source at 405 nm and 450 nm. The above results suggested that these compounds performed satisfactorily exposed to sunlight, especially C5. Notably, sunlight polymerization is easy and efficient to operate, which is conducive to the development of green chemistry and makes it sustainable.

**Table 4:** The Conv of TMPTA with PIs (1×10^−5^ mol g^−1^ TMPTA) after different times of irradiation by sunlight.

Cs	30 s	60 s	300 s
C1	3	7	14
C2	26	43	53
C3	21	38	47
C4	44	55	61
C5	56	72	75
MBF	11	31	59
TPO	43	47	51

### 3D Printing

**Figure 6:**
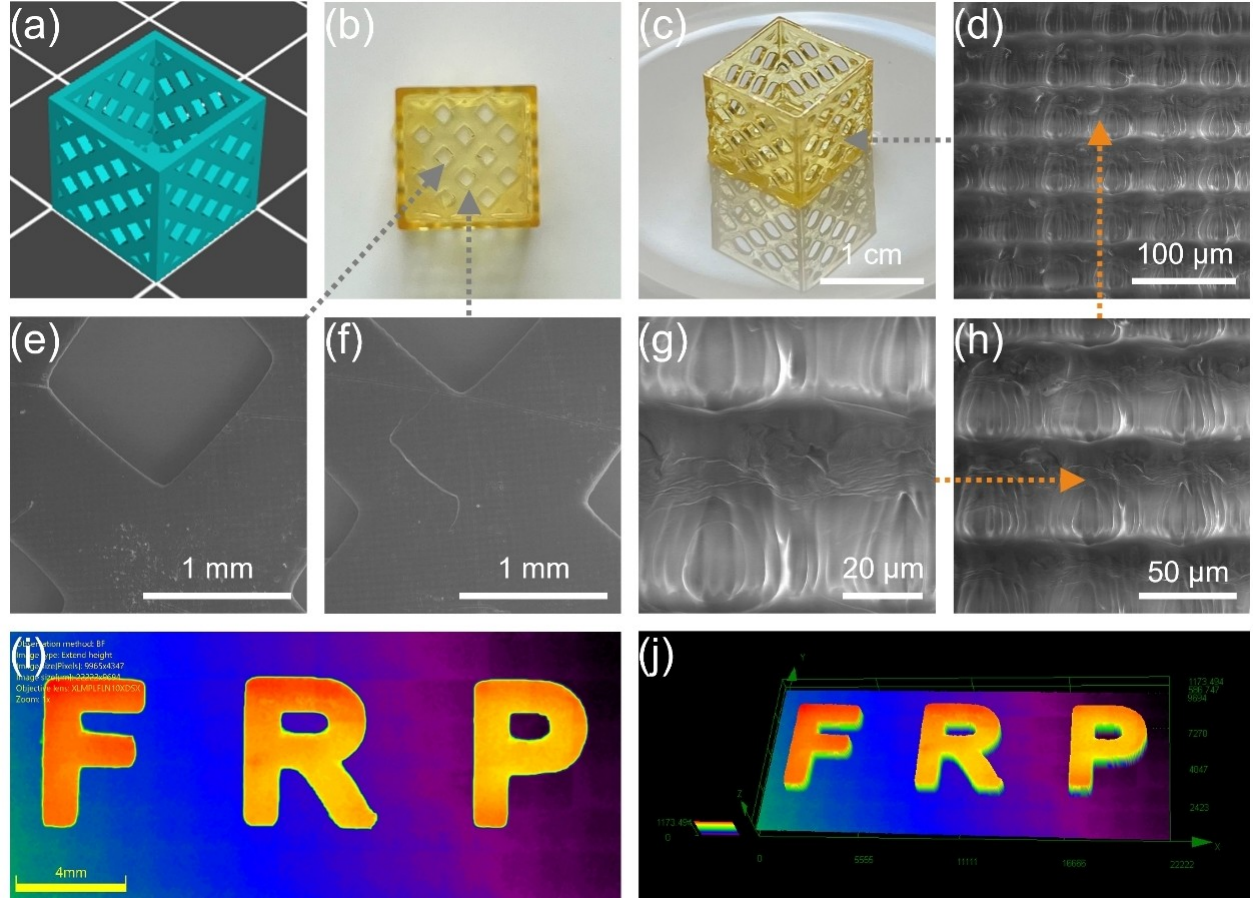
(a) 3D model; (b, c) 3D object printed with C5/TMPTA; (d, g, h) lateral view, (e, f) top view of SEM images of the 3D object printed with C5/TMPTA; (i, j) NOM images of the DLW patterns made using C5/TMPTA.

The excellent photoinitiation ability of C5 in free radical photopolymerization not only rendered it an ideal candidate for microscale and high‐precision 3D printing prototyping systems, but also was successfully applied in complex photopolymerization techniques. In this study, C5/TMPTA (1×10^−5^ mol g^−1^ TMPTA) was employed smoothly in 3D printing and direct laser write (DLW) (See Figure 6). A mesh object (10 mm×10 mm×10 mm) was printed using Digital Light Processing (DLP) light‐curing molding technology‐based 3D printer (Anycubic Photon D2 equipped with 3.2 mW cm^−2^ LED@405 nm). Dimensional accuracy is a key indicator of the quality of manufactured goods. In order to assess the structural quality of the printed object, a scanning electron microscope (SEM) was subsequently conducted to observe and characterize its surface morphology (See Figures 6a–h). The object was not coated with gold when using SEM characterization. The results showed that the surface morphology of the printed object was complete, clear, aesthetic, and displayed very high resolution (micron level). DLW was performed using a laser diode with a wavelength of 405 nm. The patterns were visualized by a numerical optical microscope (NOM) (See Figures 6i and 6j). The results revealed that the patterns could be completed not only in a short period of time, but also with excellent morphological integrity and high spatial resolution. Taken together, the above application results all confirmed that the outstanding performance of C5 in 3D printing, effectively expanding its application potential in microscale and high‐precision manufacturing, and making it suitable for application scenarios that required fast response and high resolution. Extremely remarkably, the object printed in this study not only maintained both high resolution and large print size, but the resolution of the object reached the extreme of DLP‐based printing technology, exceeding the resolution that has been reported in the literature (23 microns).[^52^, ^53^, ^54^]

### Photochemistry of Cs

#### Steady State Photolysis

**Figure 7:**
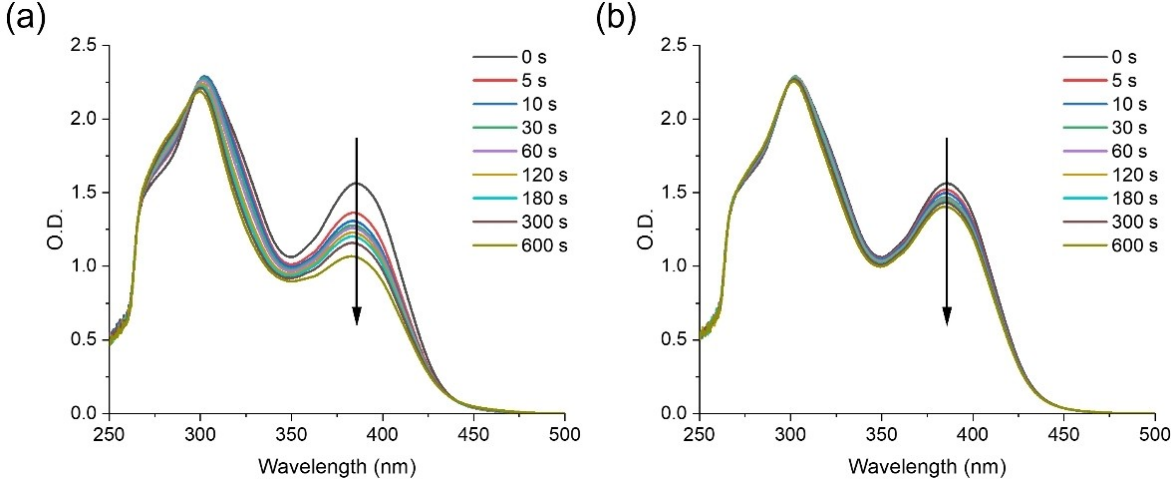
Steady state photolysis of C5 in ACN exposed to (a) LED@405 nm and (b) LED@450 nm (concentration=5×10^−5^ M).

Steady state photolysis experiments of Cs were conducted in acetonitrile (ACN) under ambient air conditions using LED@405 nm and LED@450 nm, respectively. Figure 7 showed the photolysis of C5 in acetonitrile exposed to LED@405 nm and LED@450 nm. The intensity of the absorption peaks of C5 decreased, indicating that C5 were cleaved. Notably, the degradation rate of C5 exposed to LED@405 nm was faster than that relative to its exposure to LED@450 nm. It may be due to the fact that the maximum absorption wavelength of C5 (λ_max_=386 nm) was around 405 nm wavelength and its molar extinction coefficient at 405 nm wavelength (ϵ_405_=23 200 M^−1^ cm^−1^) was larger than that at 450 nm wavelength (ϵ_450_=800 M^−1^⋅cm^−1^). This result was in coincidence with the photopolymerization kinetics obtained from C5, i.e., the initiation ability of C5 exposed to LED@405 nm was superior to that of C5 exposed to LED@450 nm. The steady state photolysis of the other Cs also showed remarkable similarity with that of C5 (See Figures S4 and S5). It is worth noting that the photobleaching was not significant, though this is not a concern for certain applications, such as adhesives and 3D printing.

#### Decarboxylation Mechanism

**Figure 8:**
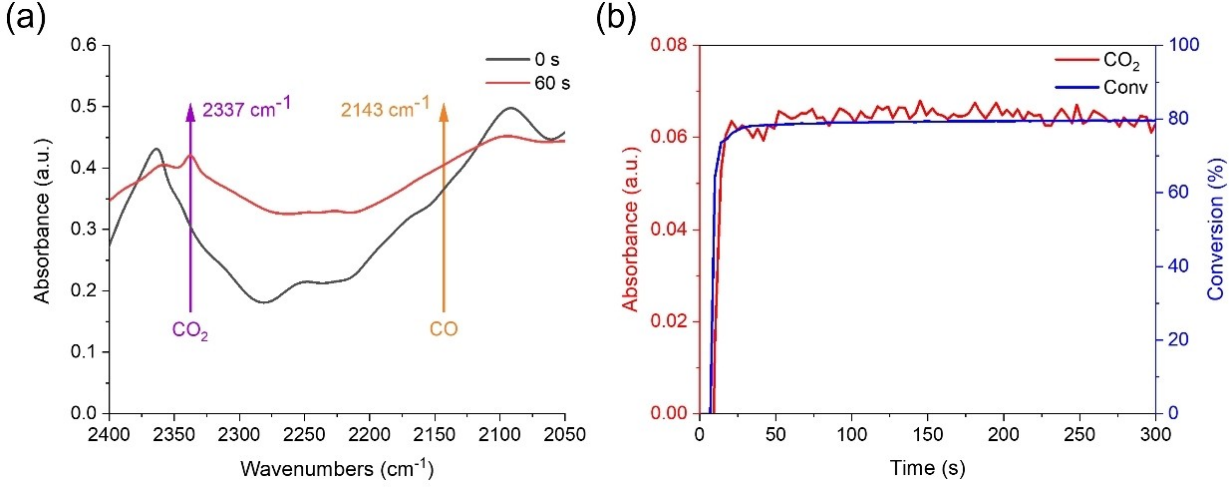
(a) Infrared spectra of C5 in TMPTA at t=0 s and 60 s; (b) The curves of absorption intensity (absorbance vs irradiation time) of CO_2_ released and functional conversions (acrylate functions conversions vs irradiation time) derived from C5/TMPTA.

CO_2_ was monitored by RT‐FTIR in photopolymerization experiments to investigate the occurrence of the decarboxylation reaction. It was effective in detecting the production of CO_2_ during the photopolymerization and thus confirming the decarboxylation reaction. C5 (1×10^−5^ mol g^−1^ TMPTA) produced a prominent new absorption peak at 2337 cm^−1^ in infrared spectra during the photopolymerization of TMPTA, which corresponded to the infrared absorption peak of CO_2_ (See Figure 8a). However, there was no new CO infrared absorption peak at 2143 cm^−1^ in infrared spectra, which suggested that the decarboxylation reaction of C5 in the photopolymerization process produced CO_2_ rather than CO. The relationships between absorption intensity of CO_2_ released, acrylate function conversions, and irradiation time were then further comprehensively analyzed (See Figure 8b). When C5 was exposed to LED@405 nm from t=10 s to t=20 s, the curve showed a rapid increase in the absorption of CO_2_ during the short period of 10 s, which revealed that the decarboxylation reaction occurred rapidly and large amounts of CO_2_ were produced. After t=20 s, the absorption of CO_2_ remained constant. This trend in CO_2_ changes observed in the decarboxylation reaction was in accordance with those observed for acrylate function conversions in the photopolymerization kinetics of TMPTA.

It can be concluded from the literature that the CO_2_ produced from C5 may be derived from the cleavage of the C−C bond between the dicarbonyl groups and the C−O bond between the ethoxy groups in the glyoxylate moiety,^[30]^ as well as the breakage of the oxime ester moiety.^[55]^ Remarkably, the occurrence of the decarboxylation reaction varied depending on the structure and initiation properties of the Cs. For TMPTA photopolymerization reactions, except for C1 (containing neither glyoxylate nor oxime ester moiety), all the other Cs underwent decarboxylation reaction to produce CO_2_. Notably, the amount of CO_2_ generated from C5 and C4 (containing both glyoxylate and oxime ester moieties) was more than that produced from C2 and C3 (containing only oxime ester moiety) (See Figure S6). A previous study showed that the CEG‐3 compound containing only glyoxylate can also generate CO_2_ (See Figures 4c and 4d),^[30]^ but its CO_2_ production was lower than that of C5. It can be clearly observed that more CO_2_ was detected in C5 than in CEG‐3 exposed to LED@405 nm. Summarizing these results, it can be determined that both the oxime ester and glyoxylate groups in C5 experienced decarboxylation reactions. In order to obtain more explicit and clearer photoinitiation mechanism, a further experiment, i.e., electron spin resonance‐spin trapping (ESR‐ST), was carried out and cross‐validated with molecular modeling.

#### ESR‐ST Experiments

**Figure 9:**
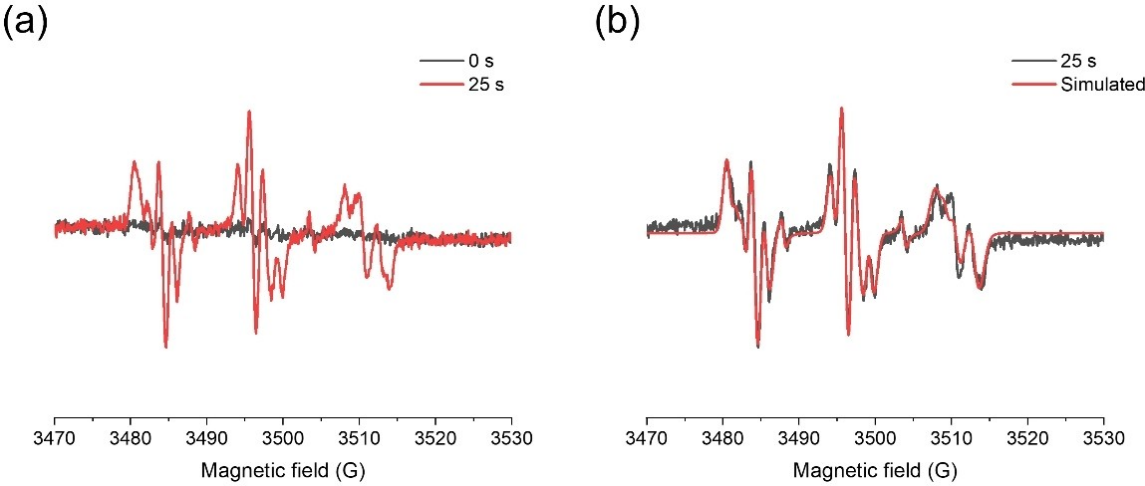
(a, b) ESR spectra of the radical adducts of C5 in N_2_ ambient condition exposed to LED@405 nm captured by PBN in *tert*‐butylbenzene.

Figure 9 shows the ESR spectra of C5 under LED@405 nm irradiation using *n‐tert*‐butyl‐α‐phenylnitrone (PBN) as the radical trapping agent. Three radical adducts of C5 were detected in the ESR‐ST experiment with hyperfine coupling constants (hfcs) of aN=13.7 G and aH=4.8 G, aN=14.0 G and aH=2.0 G, and aN=13.2 G and aH=1.3 G, which corresponded to carbon centered radicals and oxygen centered radicals in the oxime ester moiety and carbon centered radical in the glyoxylate moiety, respectively. Combined with the CO_2_ detected in the decarboxylation reaction of C5, it can be shown that both the oxime ester and the glyoxylate moieties in C5 produced CO_2_. The C−C bond between the dicarbonyl groups and the C−O bond between the ethoxy groups in the glyoxylate moiety underwent breakage under LED@405 nm irradiation. All these above results were in complete agreement with the Type I initiator behavior of C5.

#### Photoinitiation Mechanisms

According to the literature,^[30]^ the singlet state energy (E_S1_) of C5 and other Cs were determined by normalizing the intersection of the UV/Visible absorption and fluorescence spectra, as shown in Figure S7. In view of the BDE of the N−O and C−C bonds in oxime ester moiety and the C−C bond in glyoxylate moiety, and the triplet state energy (E_T1_) assessed from molecular modeling, and taking into account the possible order of the cleavage process, it can be inferred that the N−O bond is cleaved first. It may be due to the fact that the BDE of the N−O bond (BDE=43.0 kcal/mol) in oxime ester moiety was much lower than that of the C−C bond (BDE=78.5 kcal/mol) in glyoxylate moiety and the C−C bond (BDE=76.8 kcal/mol) in oxime ester moiety, and its BDE was also lower than the triplet state energy. The enthalpy of cleavage of the chemical bond in both the oxime ester (ΔH_cleavage S1_ of N−O and C−C bonds) moiety and the glyoxylate (ΔH_cleavage S1_ of C−C bond) moiety in the singlet state showed lower than that of their corresponding triplet state (ΔH_cleavage T1_) (See Tables 5 and 6), suggesting that the cleavage of the chemical bond preferred the singlet state, which reconfirmed that C5 could be used as a Type I photoinitiator. Although the ΔH_cleavage S1_ of C−C bond in the oxime ester moiety and C−C bond in the glyoxylate moiety were positive in the singlet state, the cleavage reaction would still occur if decarboxylation favored the process.

**Table 5:** Bond Dissociation Energies (BDE) of N−O and C−C bonds in the oxime ester moiety, E_S1_, E_T_, ΔH_cleavage S1_, and ΔH_cleavage T1_ of C5, ΔH_decarboxylation_ of ⋅OC(=O)C(CH_3_)_3_.

N−O BDE (kcal/mol)	E_S1_ (kcal/mol)	E_T1_ (kcal/mol)	ΔH_cleavage S1_ (kcal/mol)	ΔH_cleavage T1_ (kcal/mol)	ΔH_decarboxylation_ (kcal/mol)
43.0	65.3	49.7	−22.3	−6.7	−21.4

C−C BDE (kcal/mol)	E_S1_ (kcal/mol)	E_T1_ (kcal/mol)	ΔH_cleavage S1_ (kcal/mol)	ΔH_cleavage T1_ (kcal/mol)	ΔH_decarboxylation_ (kcal/mol)
76.8	65.3	49.7	11.5	27.1	−21.4

**Table 6:** Bond Dissociation Energies (BDE) of C−C bond in the glyoxylate moiety, E_S1_, E_T_, ΔH_cleavage S1_, and ΔH_cleavage T1_ of C5, ΔH_decarboxylation_ of ⋅C(=O)OEt.

C−C BDE (kcal/mol)	E_S1_ (kcal/mol)	E_T1_ (kcal/mol)	ΔH_cleavage S1_ (kcal/mol)	ΔH_cleavage T1_ (kcal/mol)	ΔH_decarboxylation_ (kcal/mol)
78.5	65.3	49.7	13.2	28.8	−15.1

Considered in conjunction with the results of photolysis, decarboxylation, and ESR‐ST, it would be noticeable that C5 produced free radicals for photopolymerization of acrylate monomers exposed to the LED@405 nm. Additionally, the fluorescence lifetime of C5 was less than the response time of the instrument (1.4 ns) (See Table S3). With such short fluorescence lifetimes, it can be indicated that cleavage of the excited state was fast and efficient, which usually contributed to the rapid initiation of the reaction, corresponding to its better cleavage ability from singlet excited state.

**Scheme 4:**
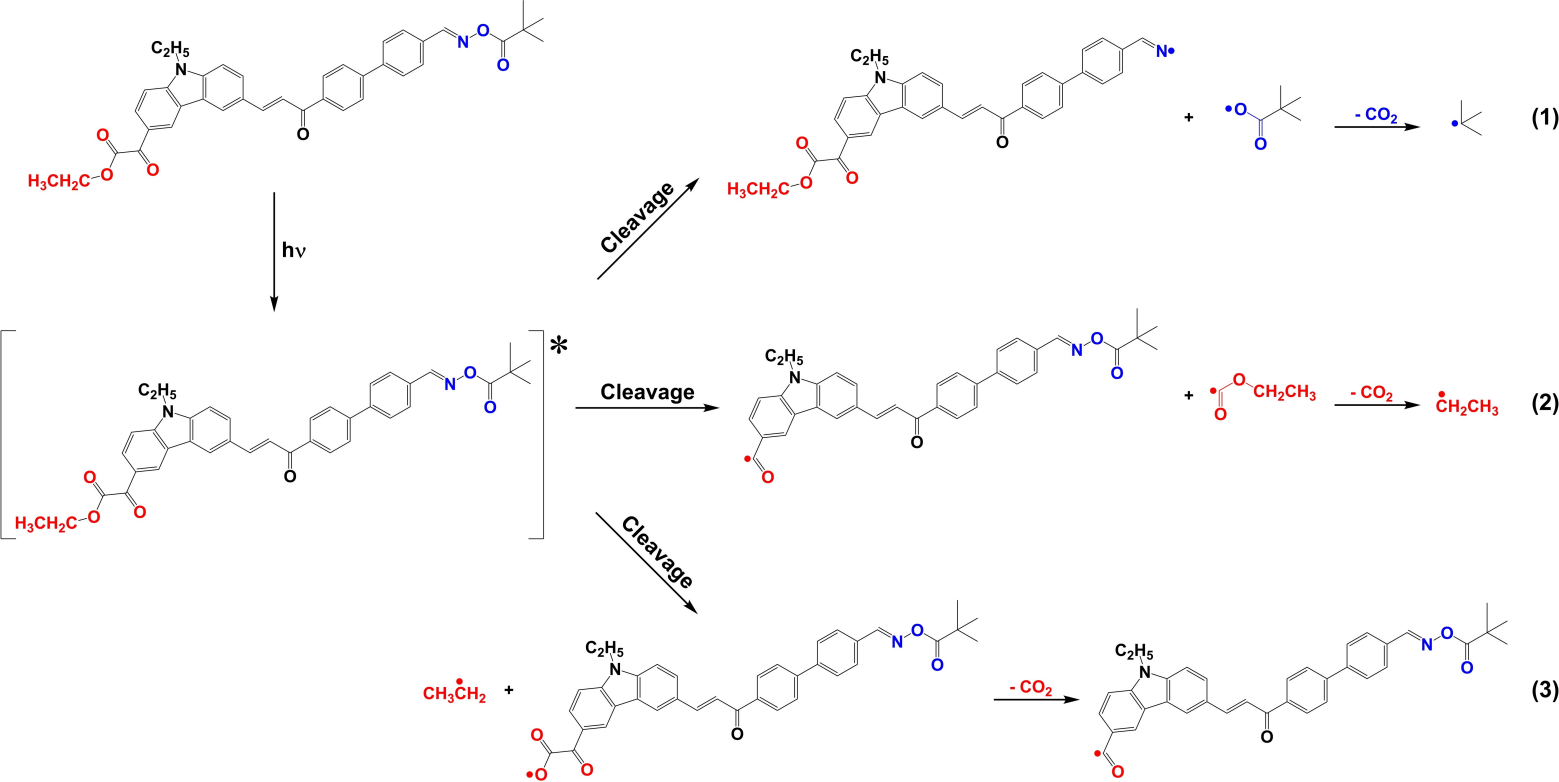
Proposed photoinitiation mechanisms of C5.

As shown in Scheme 4, the photoinitiation mechanism of C5 was proposed based on the above results. C5 generated radicals in three ways (See (1), (2), and (3) in Scheme 4) exposed to LED@405 nm. Moreover, both the oxime ester and the glyoxylate would undergo decarboxylation reaction to produce CO_2_ and free radicals that can initiate polymerization reaction of the acrylate monomer. Specifically, the N−O and C−C bonds in oxime ester R1 (R1, R2, and R3 represent the oxime ester group, the glyoxylate group, and the remaining structure in C5) can be cleaved to produce CO_2_ and ⋅C(CH_3_)_3_. The C−C bond between the dicarbonyl groups and the C−O bond between the ethoxy group in glyoxylate R2 would be cleaved. Furthermore, both pathways 2 and 3 experienced decarboxylation reactions, yielding ⋅CH_2_CH_3_ and ⋅C(=O)R_3_R_2_. The free radicals produced in all three pathways showed the ability to initiate polymerization in the presence of acrylate monomers. In view of the slightly different and less pronounced trends (conversion, Table 3 and Table 4) in systems C2−C4, it can be assumed that a positive interaction between the different paths can be observed for C5−C3 compared to compounds without glyoxylate.

### Chemical Structures/Photoinitiation Performance Relationship

Compounds containing large conjugated groups exhibited low energy gaps and strong absorption at 405 nm and 450 nm, enabling photopolymerization reaction to occur. The chemical structures of compounds (C2−C5), which contain oxime ester and/or glyoxylate moieties, had a slight influence on the E_S1_, E_T1_, BDE, and ΔH_cleavage S1 or T1_ of corresponding chemical bonds (N−O, C−C, and C−O bonds). The stabilization effect of the phenyl group attached to the oxime ester moiety could potentially hinder the decarboxylation process and reduce the photoinitiation ability. In contrast, C5, which contains both oxime ester and glyoxylate moieties, could undergo favorable decarboxylation reaction, promoting the generation of active free radicals. Therefore, C5 exhibited superior photoinitiation ability in the photopolymerization reaction. Furthermore, the more flexible ETPTA monomer likely improved the diffusion and solubility of the photoinitiators. Overall, photoinitiators with strong absorption and good solubility demonstrated enhanced photoinitiation ability.

### Cytotoxicity

**Figure 10:**
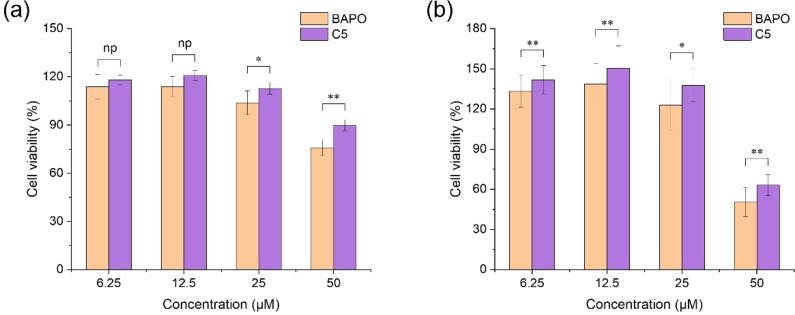
(a) Cytotoxicity of different concentrations of BAPO‐treated and C5‐treated groups in HUVECs after (a) 24 hours and (b) 48 hours of incubation. Data were shown as mean±SD. Using t‐test (*n*=3), np (p>0.05) indicates no significant difference, * (p<0.05) means significant difference, and ** (p<0.01) represents highly significant difference.

The existing TPO and phenylbis (2,4,6‐trimethylbenzoyl)‐phosphine oxide (BAPO) commercial photoinitiators have limited its application in the biomedical field due to its cytotoxicity.[^56^, ^57^] Therefore, there is a need to develop a photoinitiator that is low‐cytotoxic and highly beneficial for clinical applications. In this study, the cytotoxicity of C5 in human umbilical vein endothelial cells (HUVECs) was evaluated using the Cell Counting Kit (CCK‐8) assay with phenylbis (2,4,6‐trimethylbenzoyl)‐phosphine oxide (BAPO) as control. In the cytotoxicity assay, the cell viability of HUVECs showed an overall decreasing trend with increasing concentration of photoinitiators after 24 hours and 48 hours. However, the cell viability of C5‐treated HUVECs was consistently higher than that of BAPO‐treated HUVECs at the same concentration and for the same time. There was no significant difference in cell viability between C5‐ and BAPO‐treated groups at low concentrations (6.25 μM and 12.5 μM) after 24 hours. However, the significant difference in cell viability between C5‐ and BAPO‐treated groups was observed at high concentration (50 μM) (See Figure 10a). Particularly, the difference in cell viability between C5‐ and BAPO‐treated groups appeared to be significant after 48 hours at low and high concentrations (See Figure 10b). These above results suggested that C5 showed lower cytotoxicity in HUVECs compared to BAPO under the same conditions, especially at higher concentration and longer treatment time.

**Figure 11:**
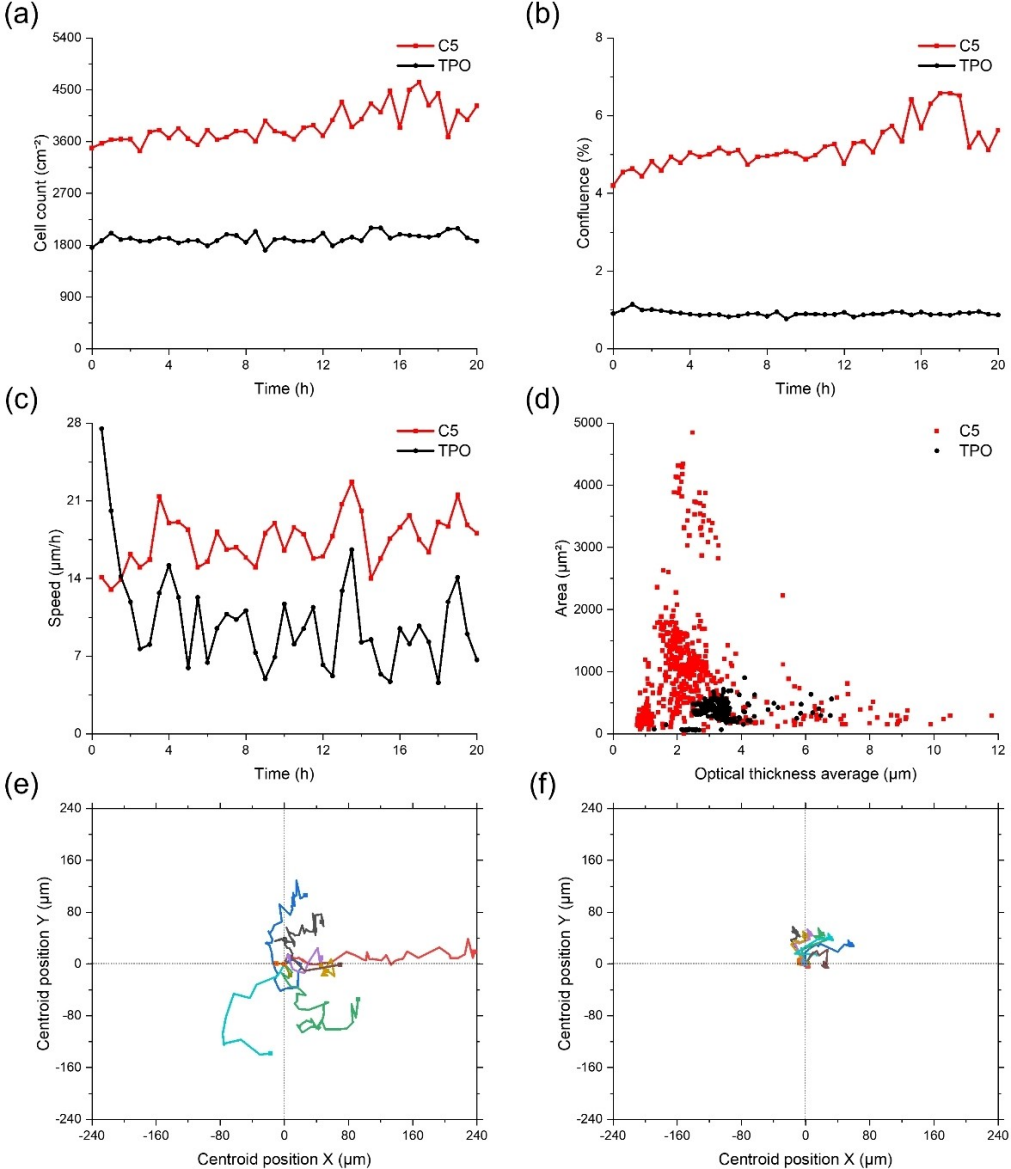
Comparison of C5 and TPO cytotoxicity on C3H10 T1/2 cells. Changes in (a) cell number, (b) cell confluency, (c) migration speed, (d) cell morphology over time were monitored and single cell tracking analysis for (e) C5 and (f) TPO were performed.

In addition, the cytotoxicity assessments of the novel C5 Type I photoinitiator on the C3H10 T1/2 cell lines were investigated by cell kinetic analysis method using the Holomonitor®M4 device with TPO as a control. The results of the kinetic dose response assays showed that the count of cells per well in the TPO treated group hardly changed substantially during the experiment period of 20 hours, indicating that the inhibitory effect of TPO on cell proliferation existed. In contrast, the number of cells per well in the C5 treated group increased significantly, indicating that cell proliferation occurred and cells maintained high viability after C5 treatment (See Figure 11a). In addition, the C5 treated group showed stable cell morphology, active migratory behavior, and clear and continuous cell movement trajectories, which further supported the positive effect of C5 on cell proliferation (See Figure S8 and Video S1). The cell confluence of the TPO treated group decreased within 20 h, indicating the inhibitory effect of TPO on cell proliferation, as well as accompanied by cell apoptosis. In comparison, the increasing cell confluence of C5 treated group (See Figure 11b) reflected the active cell proliferation, evidencing that C5 was low‐cytotoxic under the tested concentrations. The kinetic motility assays further showed that the cell migration speed of the TPO treated group was significantly slowed down, indicating that the cell viability was inhibited. Whereas, the cell migration speed of C5 treated group increased (See Figure 11c), proving that the cell motility and viability were effectively maintained. The results of cells morphology analysis demonstrated that most of the cells of TPO treated group went into apoptosis, while most of the cells of C5 treated group remained viable (See Figure 11d). Visual characterization of cell behavioral patterns by single cell tracking indicated that cells of C5 treated group migrated more widely (See Figures 11e and 11f), which further verified the low‐toxicity and good cellular activity of C5. In conclusion, all of the above results strongly suggested that the C5 was not only low‐cytotoxic, but also effective in promoting cell proliferation and migration, and showed high biocompatibility, which has a wide range of potential for clinical application.

## Conclusions

In this study, carbazole‐based chalcone glyoxylate oxime ester derivatives (Cs) containing both glyoxylate and oxime ester moieties, which have never been reported in the literature before, were synthesized as highly efficient type I PIs. Among these compounds, C5 exhibited high photoinitiation ability during free radical photopolymerization of TMPTA and ETPTA exposed to LED@405 nm and LED@450 nm as well as sunlight, and its photoinitiation ability was superior to that of commercial PIs MBF, TPO, and BAPO. Moreover, C5 can be used in 3D printing to manufacture 3D objects with complete morphology and high resolution (micrometer scale). It was predicted from molecular modeling that both the glyoxylate and the oxime ester would undergo decarboxylation to produce CO_2_ and free radicals for initiating the photopolymerization. The photochemical mechanism of C5 was thoroughly investigated by a combination of steady state photolysis, decarboxylation reactions, and ESR‐ST. Remarkably, the experimental results on the photochemical mechanism of C5 were consistent with the predicted results. Significantly, it was the first time to demonstrate the low‐toxicity of C5 by cytotoxicity analysis, which has never been reported in the literature. These findings not only provide new strategic directions and technological possibilities for the development of highly efficient Type I PIs, but also potentially open a way for cost reduction, environmental sustainability, and green chemistry applications in light curing processes. This study optimizes the design and performance of PIs using molecular modeling, and also presents an important theoretical basis and practical support for the application of photopolymerization technology in industrial production, environmentally friendly materials, energy‐efficient manufacturing, high‐precision 3D printing, and biomedical materials, among others.

## Author Contributions

**Tong Gao**: Conceptualization, Data curation, Formal Analysis, Investigation, Methodology, Validation, Writing – original draft; **Zheng Liu**: Investigation, Methodology, Validation, Writing – review & editing; **Jiansong Yin**: Formal Analysis, Investigation, Writing – review & editing; **Ji Feng**: Writing – review & editing; **Céline Dietlin**: Software, Writing – review & editing; **Fabrice Morlet‐Savary**: Formal Analysis, Supervision; **Michael Schmitt**: Formal Analysis, Supervision, review & editing; **Tatiana Petithory**: Writing – review & editing; **Laurent Pieuchot**: Writing – review & editing; **Jing Zhang**: Formal Analysis, Writing – review & editing; **Frédéric Dumur**: Formal Analysis, Writing – review & editing; **Jacques Lalevée**: Funding acquisition, Project administration, Resources, Supervision, Validation, Writing – review & editing; **Pu Xiao**: Supervision, Formal Analysis, Writing – review & editing.

## Conflict of Interests

The authors declare no conflict of interest.

1

## Supporting information

As a service to our authors and readers, this journal provides supporting information supplied by the authors. Such materials are peer reviewed and may be re‐organized for online delivery, but are not copy‐edited or typeset. Technical support issues arising from supporting information (other than missing files) should be addressed to the authors.

Supporting Information

## Data Availability

The data that support the findings of this study are available from the corresponding author upon reasonable request.
