# Supplementary material for: Novel Low‐Cytotoxic and Highly Efficient Type I Photoinitiators for Visible LED‐/Sunlight‐Induced Photopolymerization and High‐Precision 3D Printing
Source: Angew Chem Int Ed Engl. 2025 Feb 26;64(18):e202425598. doi: 10.1002/anie.202425598 (PMC12036817; doi:10.1002/anie.202425598)

Angewandte Chemie
Eine Zeitschrift der Gesellschaft Deutscher Chemiker

Supporting Information

Novel Low-Cytotoxic and Highly Efficient Type I Photoinitiators for Visible LED-/Sunlight-Induced Photopolymerization and High-Precision 3D Printing

T. Gao, Z. Liu, J. Yin, J. Feng, C. Dietlin, F. Morlet-Savary, M. Schmitt, T. Petithory, L. Pieuchot, J. Zhang, F. Dumur, J. Lalevée*, P. Xiao**

Novel Low-Cytotoxic and Highly Efficient Type I Photoinitiators for Visible LED-/Sunlight-Induced Photopolymerization and High-Precision 3D Printing

Tong Gao [a,b], Zheng Liu [c], Jiansong Yin [a,b], Ji Feng [a,b], Céline Dietlin [a,b], Fabrice Morlet-Savary [a,b], Michael Schmitt [a,b], Tatiana Petithory [a,b], Laurent Pieuchot [a,b], Jing Zhang [d], Frédéric Dumur [c]*, Jacques Lalevée [a,b]*, and Pu Xiao [e]*

[a] Université de Haute-Alsace, CNRS, IS2M UMR7361, F-68100 Mulhouse, France.
[b] Université de Strasbourg, France.
[c] Aix Marseille Univ, CNRS, ICR UMR 7273, F-13397 Marseille, France.
[d] Future Industries Institute, University of South Australia, Mawson Lakes, SA 5095, Australia.
[e] State Key Laboratory of High Performance Ceramics and Superfine Microstructure, Shanghai Institute of Ceramics, Chinese Academy of Sciences, Shanghai 200050, P. R. China.

E-mail address: jacques.lalevee@uha.fr (JL); frederic.dumur@univ-amu.fr (FD); p.xiao@mail.sic.ac.cn (PX)

Supporting Information

Table of Contents

1. Experimental Sections S2
2. Supplementary Figures S5
3. Supplementary Tables S13
4. General Informations S14

1. Experimental Section

1.1 Materials

Diphenyl (2,4,6-trimethylbenzoyl) phosphine oxide (TPO), trimethylolpropane triacrylate (TMPTA), and ethoxylated trimethylolpropane triacrylate (ETPTA) were procured from Sartomer (Arkema). The storage inhibitors were not removed. Methyl benzoylformate (MBF), acetonitrile (ACN), *tert*-butylbenzene, and the radical trapping agent *n*-*tert*-butyl-α-phenylnitrone (PBN) were obtained from Sigma-Aldrich.

1.2 Computational procedures

Computational chemistry calculations of Cs were conducted using the Gaussian 09 software package. Optimization was carried out at the B3LYP/6-31G* level.

1.3 Synthesis of Cs

The synthesis protocol for obtaining Cs with different chemical structures is shown in Scheme 2. Detailed synthetic routes and characterizations for the Cs are provided in the Supporting Information.

2.4 Photophysical property and steady state photolysis experiments

The UV-visible absorption and steady state photolysis of Cs (at a concentration of 5×10^{-5} M) in acetonitrile were investigated using JASCO V730 UV-visible spectrometer equipped with LED@405 nm (110 mW·cm^{-2}) and LED@450 nm (50 mW·cm^{-2}) light sources.

1.5 Free radical photopolymerization experiments

The photopolymerization reaction kinetics of Cs/TMPTA (at a concentration of 1×10^{-5} mol·g^{-1} in TMPTA) and Cs/ETPTA (at a concentration of 1×10^{-5} mol·g^{-1} and 1×10^{-6} mol·g^{-1} in ETPTA) in laminate between two polypropylene films were evaluated using JASCO FTIR-6600 spectrometer equipped with LED@405 nm and LED@450 nm. The function conversions (Conv) of acrylate monomers were calculated by monitoring the signal area at different time approximately about 1620 cm^{-1}. The following equation was used to calculate the Conv:

$$Conv(\%) = \frac{A_0 - A_t}{A_0} \times 100\%$$

where A_0 represents for the peak area at 0 s, and A_t indicates the peak area at t s.

1.6 Fluorescence property experiments

The fluorescence properties of Cs (at a concentration of 5×10^{-5} M) in acetonitrile were studied using JASCO FP-6200 spectrofluorometer. Subsequently, the

fluorescence excited state lifetimes of Cs were determined with the assistance of the time-correlated single-photon counting system HORIBA® DeltaFlex and the HORIBA® PPD-850 detector. The impulse response function (IRF) of the instrument was evaluated by means of a colloidal silica suspension LUDOX. The excitation wavelength was 367 nm and the pulse duration was minimized to 1.4 ns.

1.7 Electron spin resonance spin trapping (ESR-ST) experiments

The free radical detection of Cs (at a concentration of 1×10^{-4} M) was carried out using X-band spectrometer (Bruker EMX-Plus) equipped with LED@405 nm at room temperature under N_2 atmosphere. PBN (5×10^{-4} M) and *tert*-butylbenzene were used as radical trapping agent and solvent. And the ESR spectra were simulated using the PEST WINSIM program.

1.8 3D printing experiments

The 3D printing of Cs/TMPTA was conducted with 3D printer (Anycubic Photon D2 equipped with 3.2 $mW\cdot cm^{-2}$ LED@405 nm) based on Digital Light Processing (DLP) light-curing molding technology. Afterwards, the 3D printed object was observed by scanning electron microscope (SEM, FEI QUANTA 400) and numerical optical microscope (NOM, OLYMPUS DSX-HRSU). The object was not coated with gold when using SEM characterization. The direct laser write experiments (DLW) was performed employing a 405 nm laser diode. Subsequently, the patterns obtained from the DLW were observed by NOM.

1.9 Cytotoxicity assays

The cytotoxicity of C5 in human umbilical vein endothelial cells (HUVECs) was evaluated using the Cell Counting Kit (CCK-8) assay with phenylbis (2,4,6-trimethylbenzoyl)-phosphine oxide (BAPO) as control. A 1 mM solution of photoinitiators was prepared with dimethyl sulfoxide (DMSO). Then, it was diluted with Dulbecco's modified Eagle's medium/F12 (DMEM/F12) containing 10 % fetal bovine serum (FBS) to different concentrations (12.5, 25, 50 and 100 μM). HUVECs were seeded in DMEM/F12 containing 10 % FBS at a density of 1×10^4 cells/well into 96-well plates and cultured overnight in humidified incubator (37 °C, 5 % CO_2). HUVECs were co-cultured with medium containing different concentrations of PIs for 24 hours and 48 hours. The cytotoxicity of photoinitiators in HUVECs was assesed using CCK-8. Experiments were conducted independently and repeated three times. Data were shown as mean ± SD. Using t-test np ($p > 0.05$) indicates no significant difference, * ($p < 0.05$) means significant difference, and ** ($p < 0.01$) represents highly significant difference. Cell viability and proliferation assays were performed using the C3H10 T1/2 cell line. Cells were seeded into 6-well plates the day before the

experiment to allow attachment at a density of 2×10^4 cells per well in Dulbecco's modified Eagle's medium (DMEM) supplemented with 10% fetal bovine serum (FBS) and incubated at 37 °C with 5% CO_2. A concentration of 50 µM was used for each photoinitiator. Immediately prior to imaging, the medium was replaced with fresh medium containing the photoinitiator at the same concentration. Cell observations were performed using a Holomonitor® M4 phase holographic microscope (PHI AB, Lund, Sweden) with a 10x phase contrast objective. The system was placed directly in the incubator at 37 °C with 5% CO_2 and no staining was required. Ten images per well were acquired every 30 minutes, allowing continuous monitoring of cell viability and proliferation over 20 hours.

1. Supplementary Figures

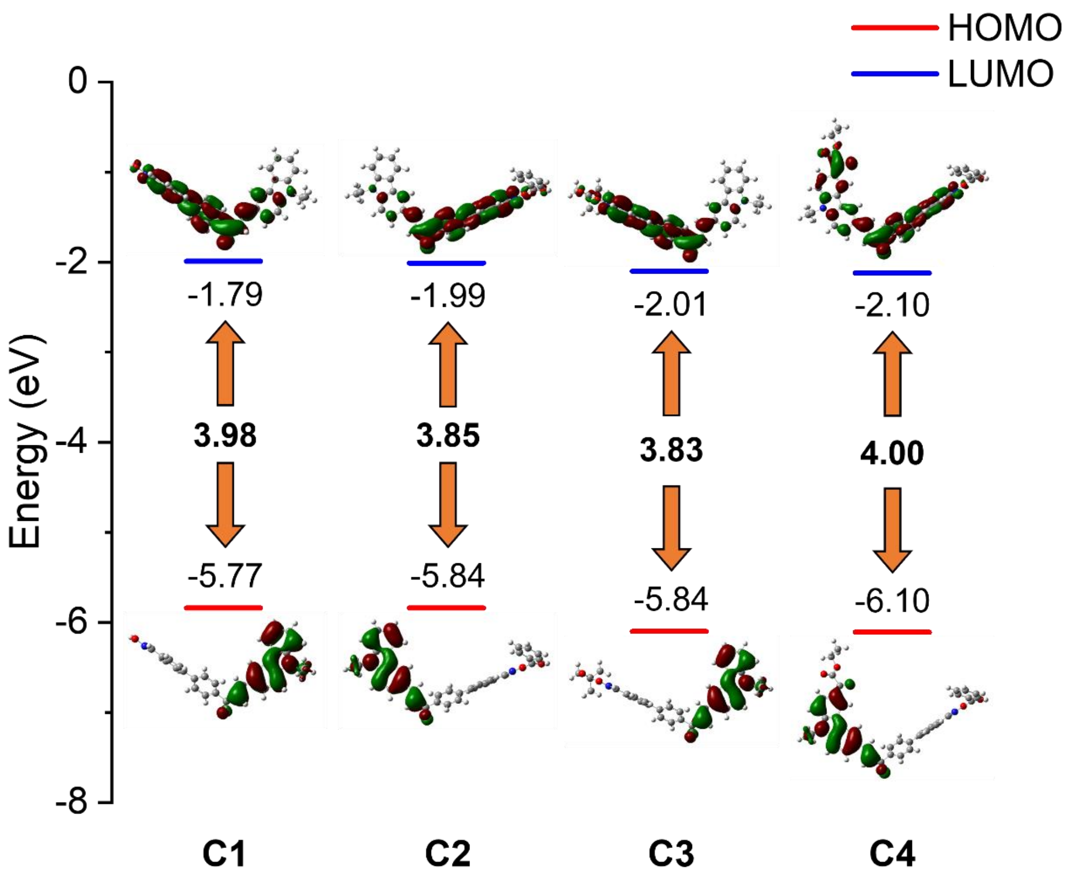

Figure S1. The HOMO, LUMO, and energy gap of C1, C2, C3, and C4. Optimization was carried out at the B3LYP/6-31G* level and orbitals were optimized at the MPW1PW91/6-31G* level of theory at a single point (isovalue = 0.02).

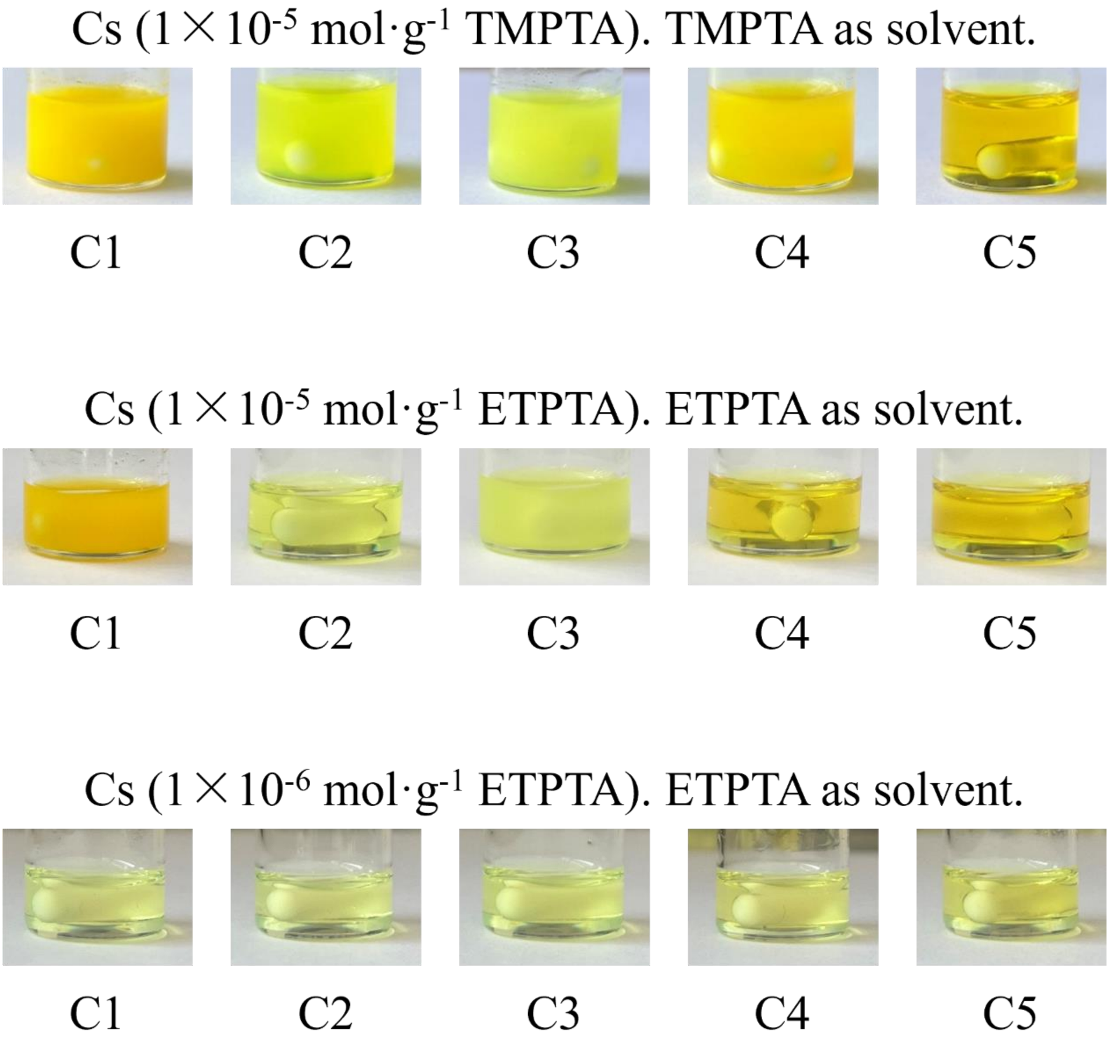

Figure S2. Solubility of Cs. TMPTA and ETPTA as solvent.

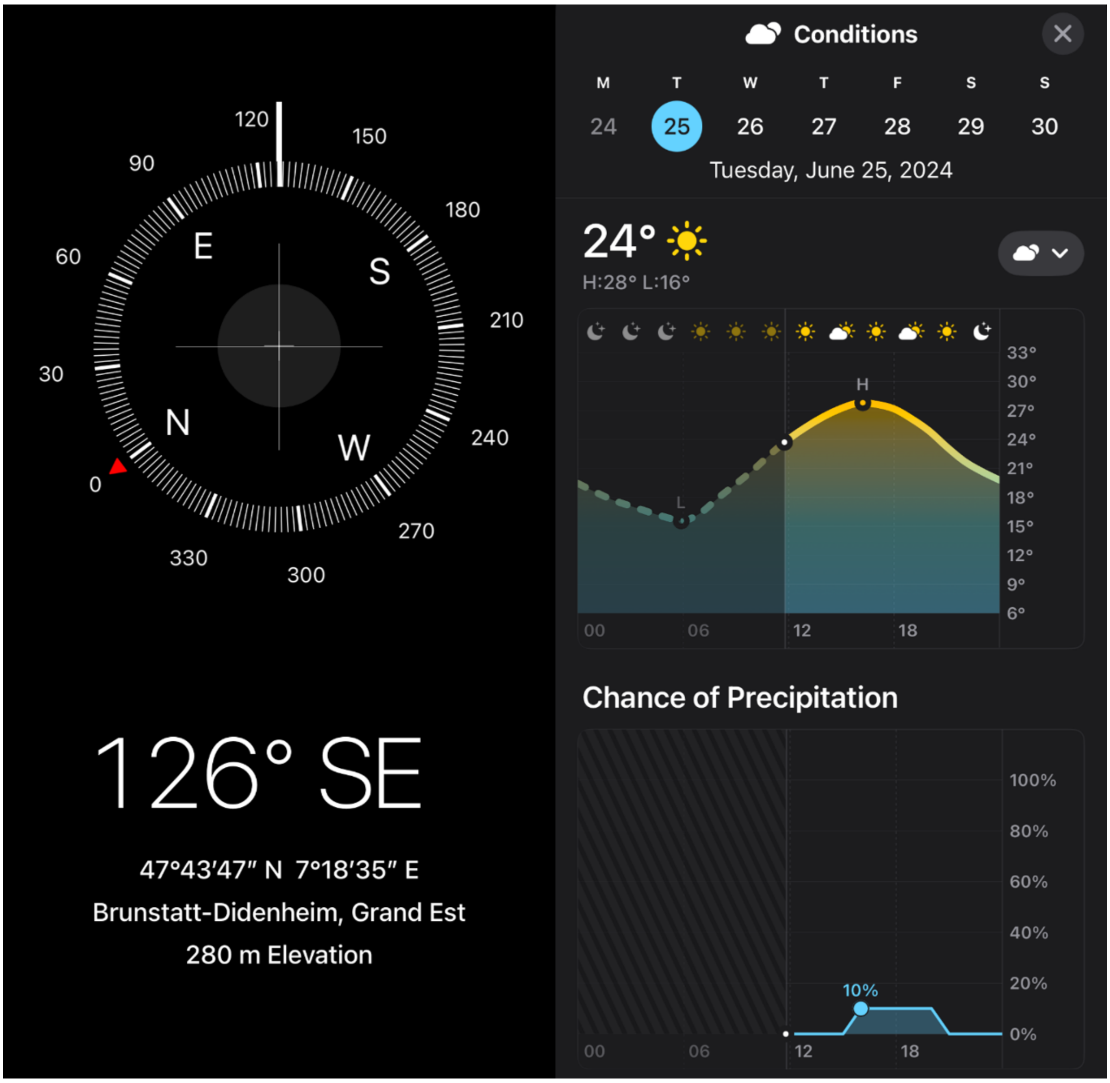

Figure S3. Environmental conditions for free radical photopolymerization kinetics of TMPTA with photoinitiators (1×10^{-5} mol·g^{-1} TMPTA) under sunlight irradiation.

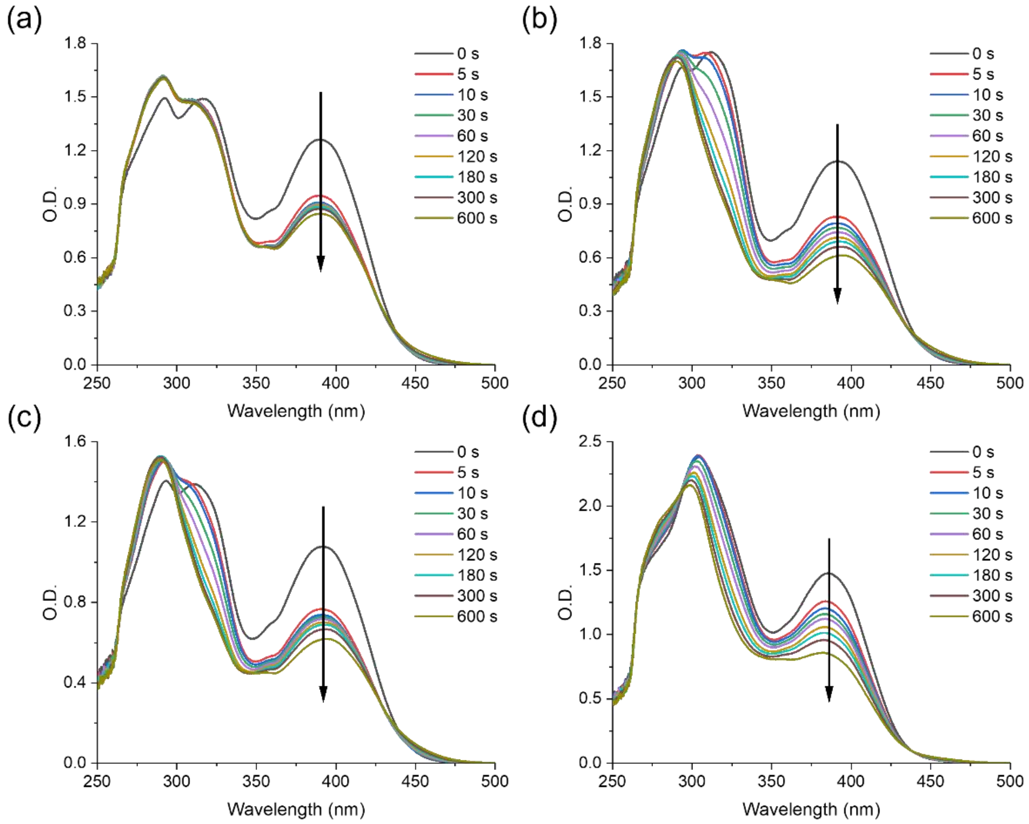

Figure S4. Steady state photolysis of (a) C1, (b) C2, (c) C3, and (d) C4 in acetonitrile exposed to LED@405 nm (concentration = 5×10^{-5} M).

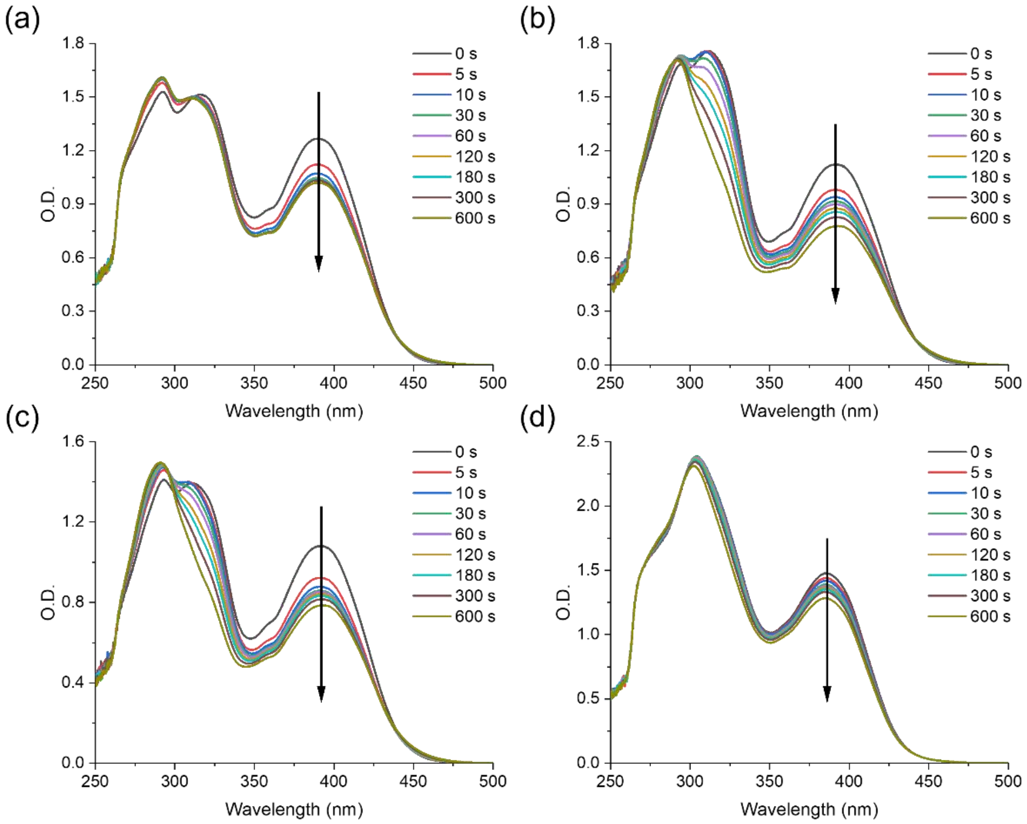

Figure S5. Steady state photolysis of (a) C1, (b) C2, (c) C3, and (d) C4 in acetonitrile exposed to 450 nm LED (concentration = 5 × 10^{-5} M).

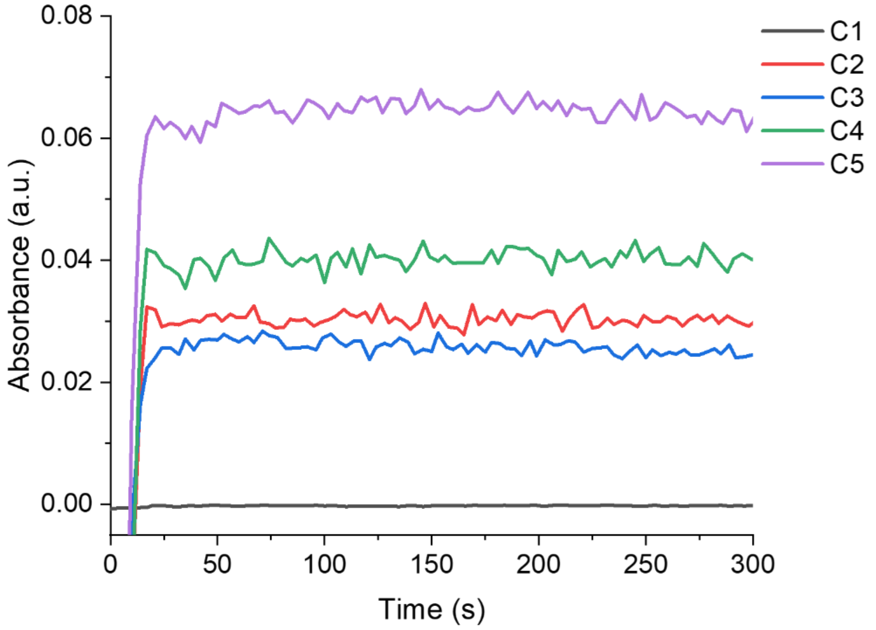

Figure S6. The curves of absorption intensity (absorbance vs irradiation time) of CO_2 derived from Cs/TMPTA. The irradiation starts at t = 10 s.

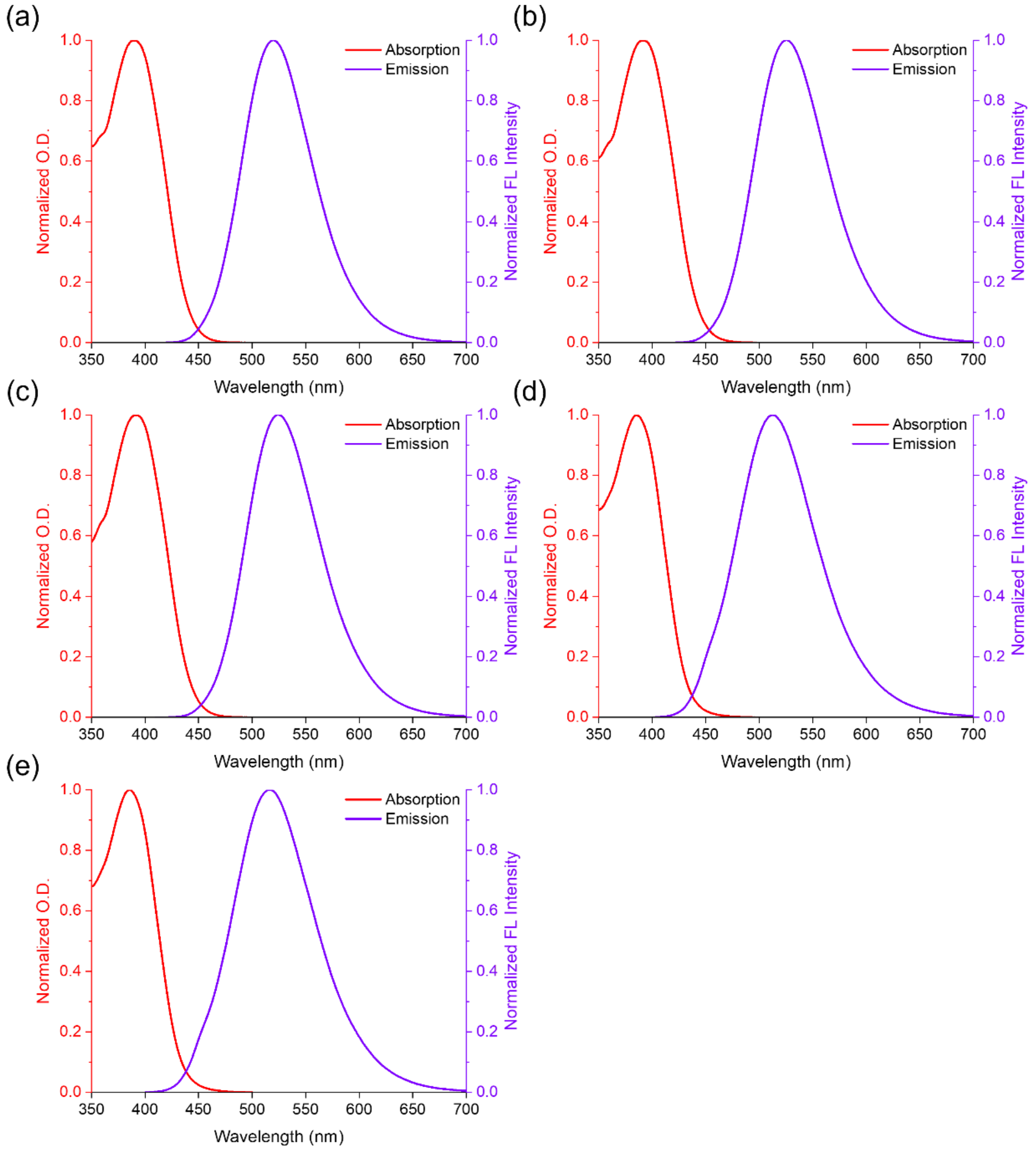

Figure S7. The curves of singlet-state energy determination of (a) C1, (b) C2, (c) C3, (d) C4, and (e) C5.

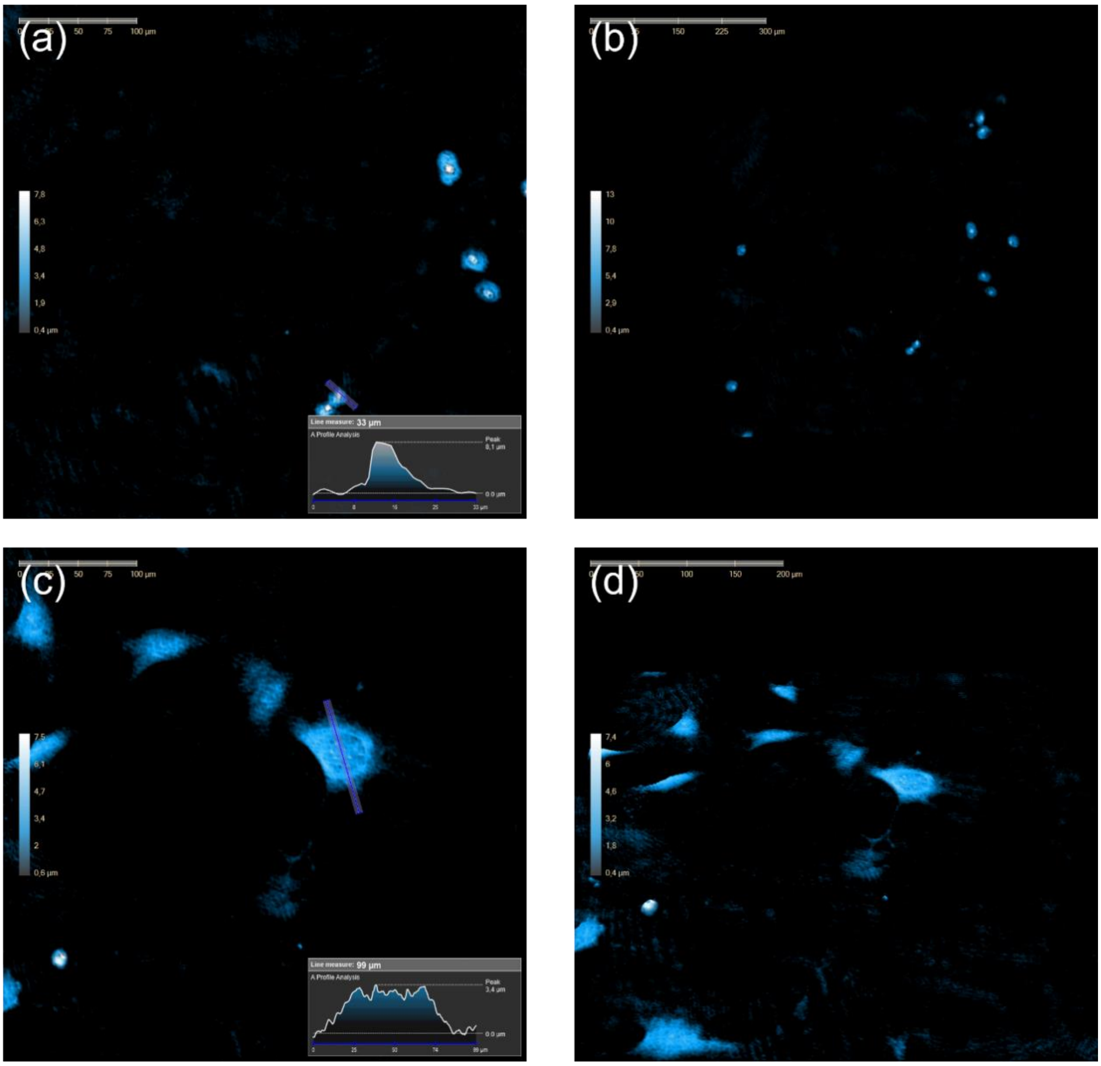

Figure S8. (a) and (b) Kinetic dose response assays images of TPO treated group; (c) and (d) kinetic dose response assays images of C5 treated group. Different locations were randomly selected for imaging. The thickness of the C3H10 T1/2 cell lines was shown by the color band represented on the left side of the figures S8(a) and S8(c). The profiles analysis was shown in the lower-right vignettes of figures S8(a) and S8(c).

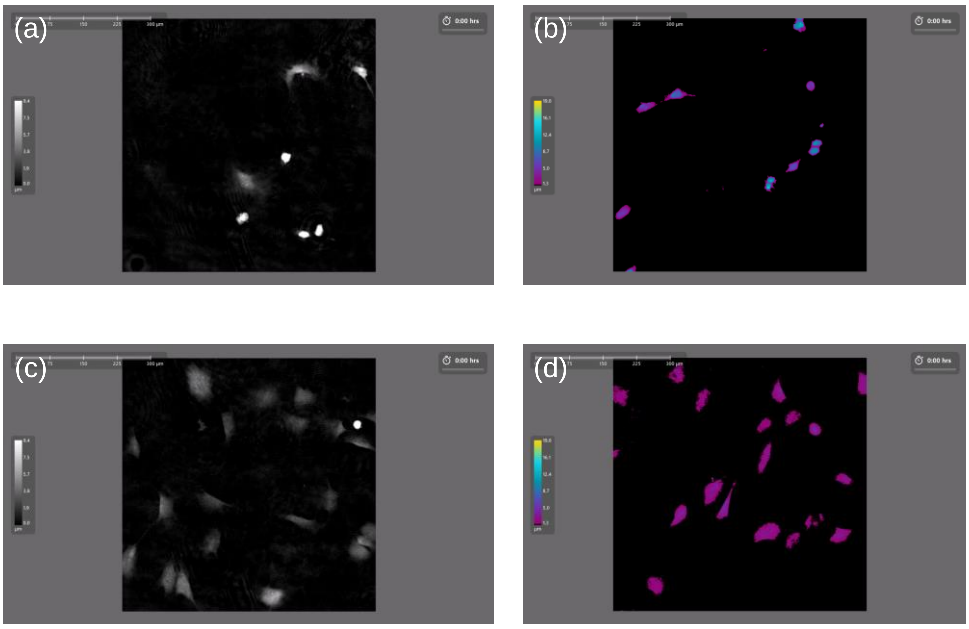

Video S1. Holomonitor captured a 4 sec time lapse of the 20 h cell observation test at a scale of 300 μm for details of (a) (black and white video) and (b) (color video) TPO treated group, and (c) (black and white video) and (d) (color video) C5 treated group.

2. Supplementary Tables

Table S1. Energy gaps of Cs.

Cs	C1	C2	C3	C4	C5
Energy gap (eV)	3.98	3.85	3.83	4.00	3.99

Table S2. Solubility of Cs in TMPTA and ETPTA.

Cs	PIs 1×10^{-5} mol·g^{-1} TMPTA	PIs 1×10^{-5} mol·g^{-1} ETPTA	PIs 1×10^{-6} mol·g^{-1} ETPTA
C1	-	-	+
C2	-	+	+
C3	-	-	+
C4	-	+	+
C5	+	+	+

Note: "+" indicates that Cs were completely dissolved; "-" indicates that Cs were not completely dissolved.

Table S3. Fluorescence lifetime of Cs in acetonitrile (concentration = 5×10^{-5} M).

Cs	C1	C2	C3	C4	C5
Lifetime (ns)	1.7	2.0	1.9	<1.4	<1.4

4. General informations

All reagents and solvents were purchased from Aldrich or Alfa Aesar and used as received without further purification. Mass spectroscopy was performed by the Spectropole of Aix-Marseille University. ESI mass spectral analyses were recorded with a 3200 QTRAP (Applied Biosystems SCIEX) mass spectrometer. The HRMS mass spectral analysis was performed with a QStar Elite (Applied Biosystems SCIEX) mass spectrometer. Elemental analyses were recorded with a Thermo Finnigan EA 1112 elemental analysis apparatus driven by the Eager 300 software. ^{1}H and ^{13}C NMR spectra were determined at room temperature in 5 mm o.d. tubes on a Bruker Avance 400 or a Bruker Avance 300 spectrometer of the Spectropole: ^{1}H (400 MHz), ^{1}H (300 MHz), ^{13}C (100 MHz), and ^{13}C (75 MHz). All ^{1}H chemical shifts were referenced to the solvent peak $CDCl_3$ (7.26 ppm), DMSO-d_6 (2.49 ppm) and the ^{13}C chemical shifts were referenced to the solvent peak $CDCl_3$ (77.16 ppm), DMSO-d_6 (39.52 ppm).

Synthesis of (*E*)-1-(4-bromophenyl)-3-(9-ethyl-9H-carbazol-3-yl)prop-2-en-1-one **(C-Br)**

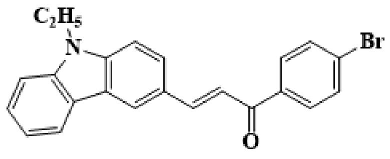

Chemical Formula: $C_{23}H_{18}BrNO$
Molecular Weight: 404.3070

9-ethyl-9H-carbazole-3-carbaldehyde (5.25 g, 23.51 mmol, M = 223.28 $g.mol^{-1}$) and 4'-bromoacetophenone (4.68 g, 23.51 mmol, M = 199.05 $g.mol^{-1}$) were dissolved in methanol (MeOH). 2.5 molar aq. KOH (158 ml) added dropwise at room temperature. After the complete addition, reaction mixture allowed to stir for 24 h. The precipitated product was filtered, washed with water by several times and dried under vacuum. The raw product was recrystallized from dichloromethane/ether to give the product as a solid (6.77 g, 71.2% yield).

^{1}H NMR (400 MHz, $CDCl_3$) δ 8.36 (s, 1H), 8.14 (d, *J* = 7.7 Hz, 1H), 8.05 (d, *J* = 15.5 Hz, 1H), 7.93 (d, *J* = 8.5 Hz, 2H), 7.78 (dd, *J* = 8.5, 1.3 Hz, 1H), 7.65 (d, *J* = 8.5 Hz, 2H), 7.54 – 7.48 (m, 2H), 7.42 (t, *J* = 8.5 Hz, 2H), 7.30 (t, *J* = 7.4 Hz, 1H), 4.37 (q, *J* = 7.2 Hz, 2H), 1.46 (t, *J* = 7.2 Hz, 3H).

^{13}C NMR (101 MHz, $CDCl_3$) δ 189.41 (s), 147.10 (s), 141.61 (s), 140.53 (s), 137.57 (s), 131.82 (s), 129.99 (s), 127.40 (s), 126.43 (s), 126.42 (s), 125.76 (s), 123.55 (s), 122.87 (s), 121.78 (s), 120.66 (s), 119.81 (s), 118.43 (s), 108.96 (s), 108.94 (s), 77.35 (s), 77.04 (s), 76.72 (s), 37.81 (s), 13.84 (s).

^{1}H NMR spectrum of (*E*)-1-(4-bromophenyl)-3-(9-ethyl-9H-carbazol-3-yl)prop-2-en-1-one **(C-Br)**

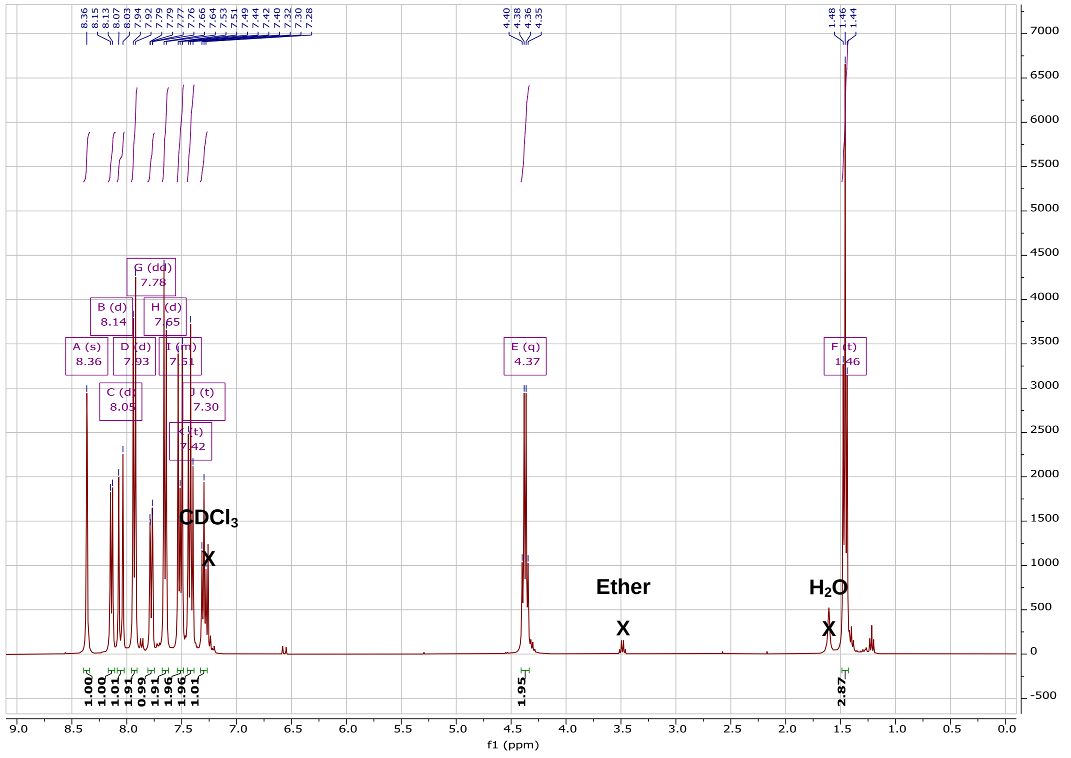

^{13}C NMR spectrum of (*E*)-1-(4-bromophenyl)-3-(9-ethyl-9H-carbazol-3-yl)prop-2-en-1-one **(C-Br)**

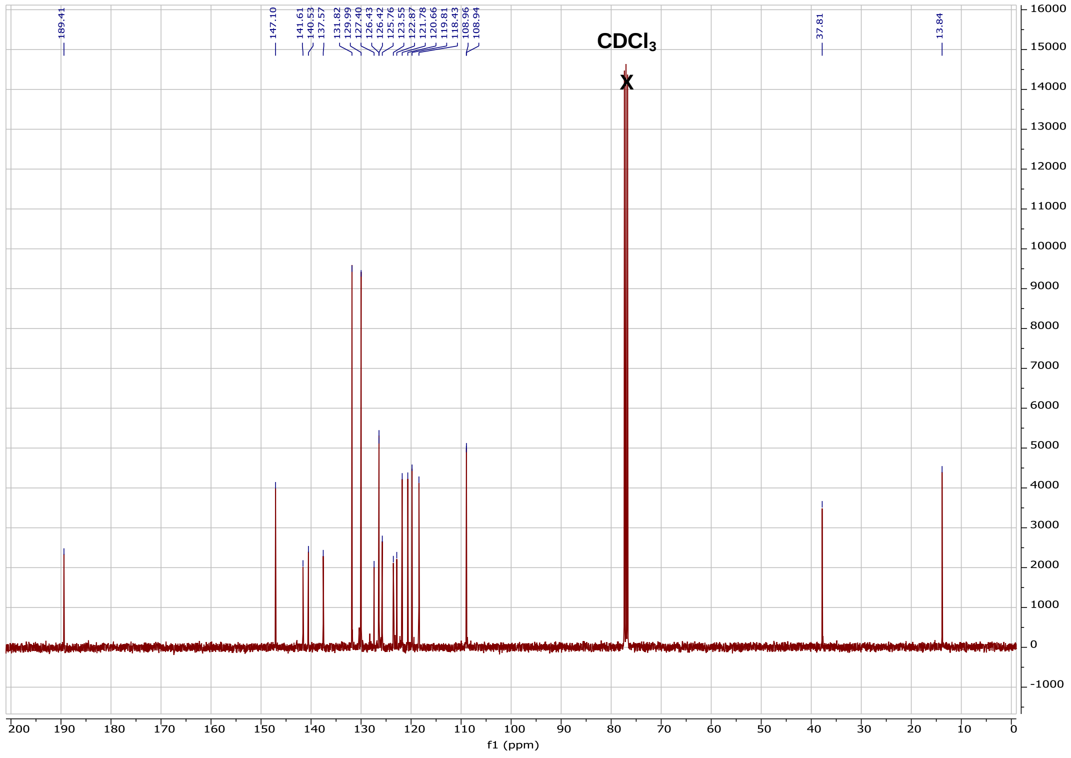

Synthesis of (*E*)-4'-(3-(9-ethyl-9H-carbazol-3-yl)acryloyl)-[1,1'-biphenyl]-4-carbaldehyde **(C0)**

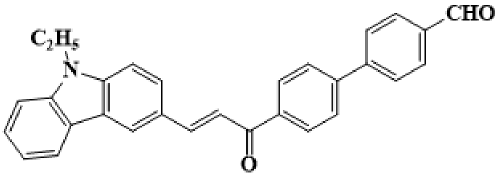

Chemical Formula: $C_{30}H_{23}NO_2$
Molecular Weight: 429.5190

Tetrakis(triphenylphosphine)palladium (0) ($Pd(PPh_3)_4$) (1.95 g, 1.69 mmol, M = 1155.56 $g.mol^{-1}$) was added to a mixture of (*E*)-1-(4-bromophenyl)-3-(9-ethyl-9H-carbazol-3-yl)prop-2-en-1-one (11.54 g, 28.54 mmol, M = 404.31 $g.mol^{-1}$), 4-formylphenylboronic acid (4.71 g, 31.40 mmol, M = 149.94 $g.mol^{-1}$), toluene (Tol) (260 mL), ethanol (EtOH) (130 mL) and an aqueous potassium carbonate (K_2CO_3) solution (2 M, 276.40 g in 1 L water, 130 mL) under vigorous stirring. The mixture was stirred at 80°C for 48 h under a nitrogen atmosphere. After cooling to room temperature, the solvent was removed under reduced pressure. The residue was extracted with DCM. The organic phase was washed several times with water, dried over $MgSO_4$ and the solvent removed under reduced pressure (11.85 g, 96.7% yield).

^1H NMR (400 MHz, $CDCl_3$) δ 10.10 – 10.06 (m, 1H), 8.39 (t, *J* = 3.5 Hz, 1H), 8.21 – 8.13 (m, 3H), 8.10 (d, *J* = 15.5 Hz, 1H), 8.01 – 7.97 (m, 2H), 7.84 – 7.75 (m, 5H), 7.64 – 7.59 (m, 1H), 7.55 – 7.51 (m, 1H), 7.43 (dd, *J* = 8.4, 4.1 Hz, 2H), 7.32 – 7.27 (m, 1H), 4.38 (q, *J* = 7.2 Hz, 2H), 1.46 (t, *J* = 7.2 Hz, 3H).

^{13}C NMR (101 MHz, $CDCl_3$) δ 191.75 (s), 189.87 (s), 146.86 (s), 145.98 (s), 143.45 (s), 141.59 (s), 140.53 (s), 138.55 (s), 135.78 (s), 130.33 (s), 129.18 (s), 127.90 (s), 127.53 (s), 126.42 (s), 125.88 (s), 123.56 (s), 122.88 (s), 121.77 (s), 120.66 (s), 119.80 (s), 118.84 (s), 108.95 (s), 77.35 (s), 77.04 (s), 76.72 (s), 37.82 (s), 13.84 (s).

^{1}H NMR spectrum of (*E*)-4'-(3-(9-ethyl-9H-carbazol-3-yl)acryloyl)-[1,1'-biphenyl]-4-carbaldehyde **(C0)**

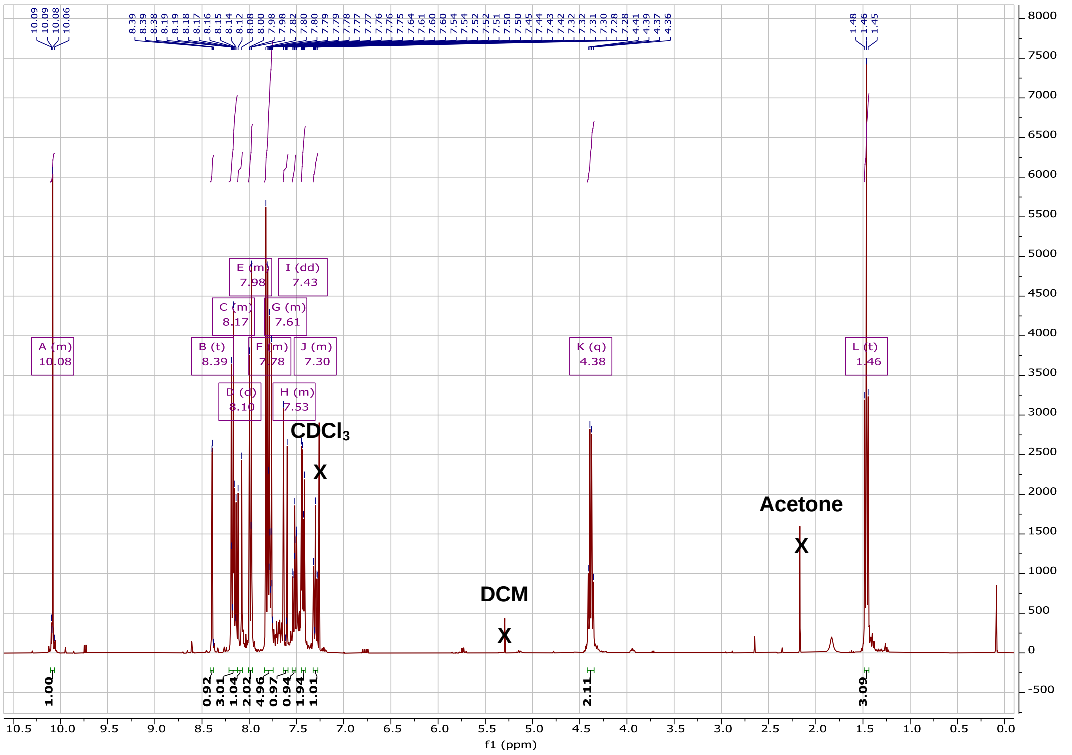

^{13}C NMR spectrum of (*E*)-4'-(3-(9-ethyl-9H-carbazol-3-yl)acryloyl)-[1,1'-biphenyl]-4-carbaldehyde **(C0)**

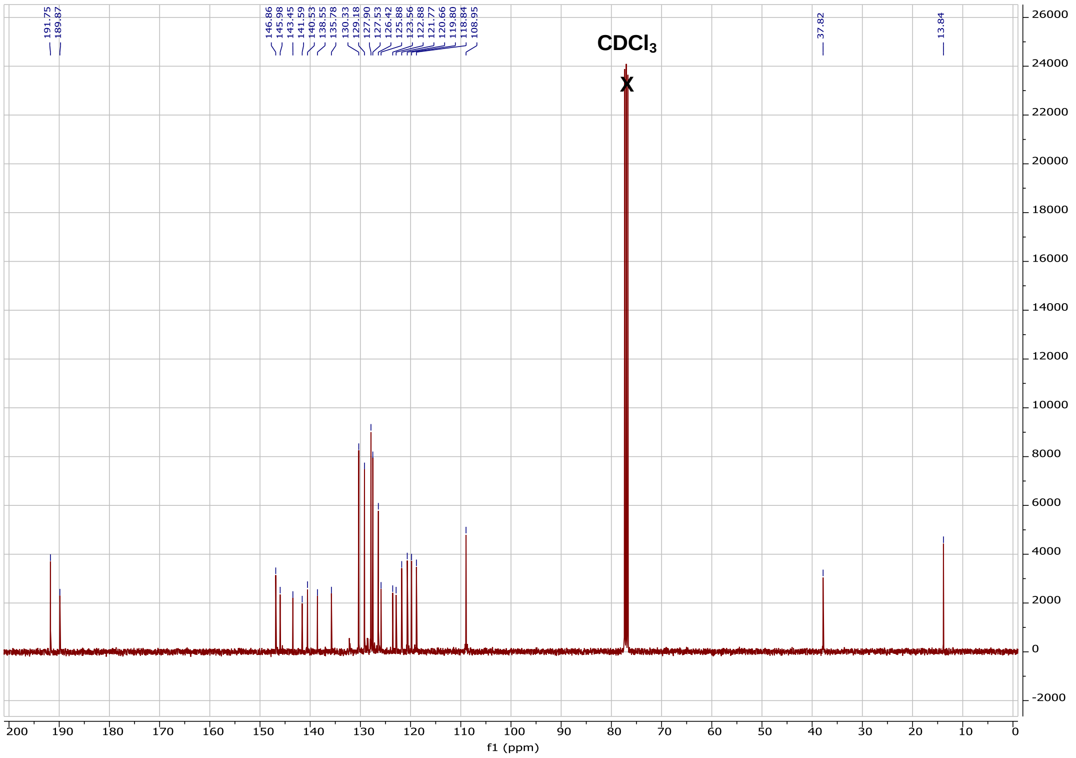

Synthesis of (*E*)-4'-((*E*)-3-(9-ethyl-9H-carbazol-3-yl)acryloyl)-[1,1'-biphenyl]-4-carbaldehyde oxime **(C1)**

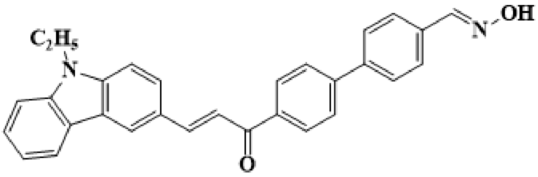

Chemical Formula: $C_{30}H_{24}N_2O_2$
Molecular Weight: 444.5340

(*E*)-4'-(3-(9-ethyl-9H-carbazol-3-yl)acryloyl)-[1,1'-biphenyl]-4-carbaldehyde (13.73 g, 31.97 mmol, M = 429.17 g/mol), hydroxylamine hydrochloride ($NH_2OH \cdot HCl$) (2.22 g, 31.97 mmol, M = 69.49 g/mol) and sodium acetate (NaAc) (2.62 g, 31.97 mmol, M = 82.03 g/mol) were dissolved in a mixture of methanol:water:THF (100 mL/100 mL/500 mL) and the solution was refluxed for 2 days. After cooling the solvent was removed under reduced pressure. The residue was washed several times with water and dried under vacuum to give the product as an orange solid (13.21 g, 93.0% yield).

^{1}H NMR (300 MHz, DMSO) δ 11.35 (d, *J* = 16.7 Hz, 1H), 8.76 (s, 1H), 8.32 – 8.26 (m, 2H), 8.23 (s, 1H), 8.02 (s, 2H), 7.92 (t, *J* = 7.0 Hz, 2H), 7.82 (t, *J* = 8.6 Hz, 2H), 7.74 (d, *J* = 8.5 Hz, 2H), 7.68 (d, *J* = 8.7 Hz, 1H), 7.65 – 7.61 (m, 1H), 7.49 (t, *J* = 6.0 Hz, 1H), 7.28 (t, *J* = 7.4 Hz, 1H), 4.46 (q, *J* = 6.8 Hz, 2H), 1.33 (dd, *J* = 11.6, 4.5 Hz, 3H).

^{13}C NMR (75 MHz, DMSO) δ 188.77 (s), 148.21 (s), 146.29 (s), 143.92 (s), 141.61 (s), 140.60 (s), 140.05 (s), 137.60 (s), 133.53 (s), 129.66 (s), 127.79 (s), 127.56 (s), 127.30 (s), 126.81 (s), 126.18 (s), 123.22 (s), 122.78 (s), 122.61 (s), 121.13 (s), 120.03 (s), 119.17 (s), 110.05 (s), 67.48 (s), 37.68 (s), 25.58 (s), 14.21 (s).

^{1}H NMR spectrum of (*E*)-4'-((*E*)-3-(9-ethyl-9H-carbazol-3-yl)acryloyl)-[1,1'-biphenyl]-4-carbaldehyde oxime **(C1)**

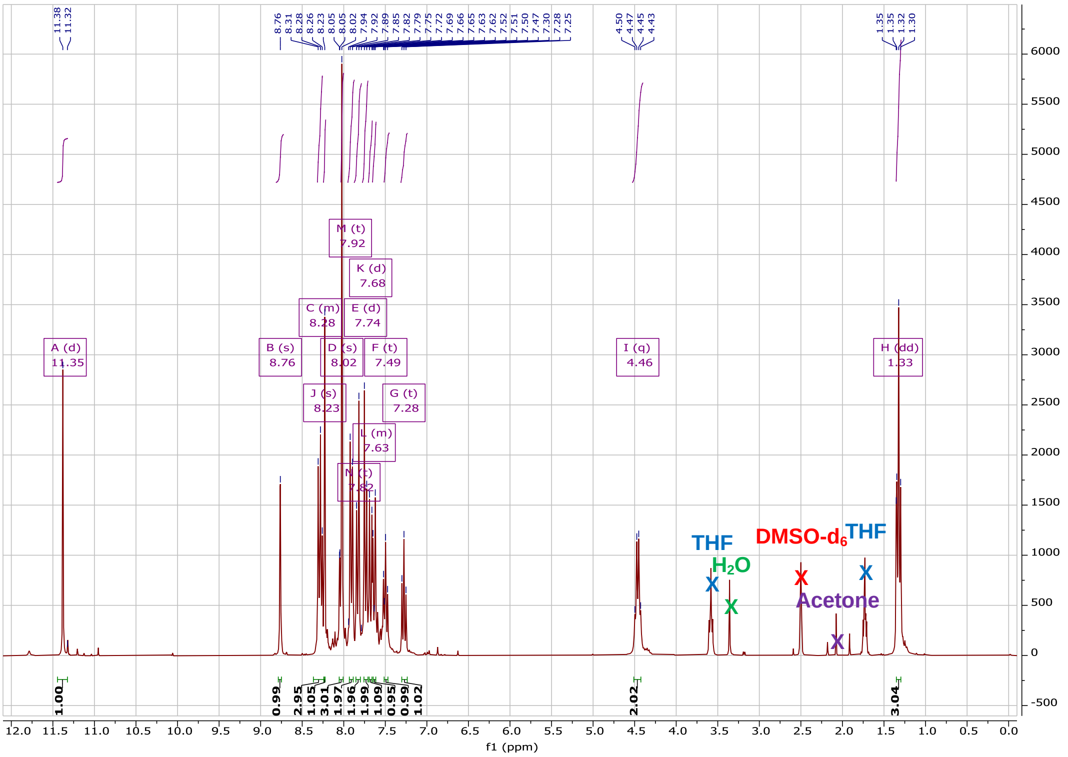

^{13}C NMR spectrum of (*E*)-4'-((*E*)-3-(9-ethyl-9H-carbazol-3-yl)acryloyl)-[1,1'-biphenyl]-4-carbaldehyde oxime **(C1)**

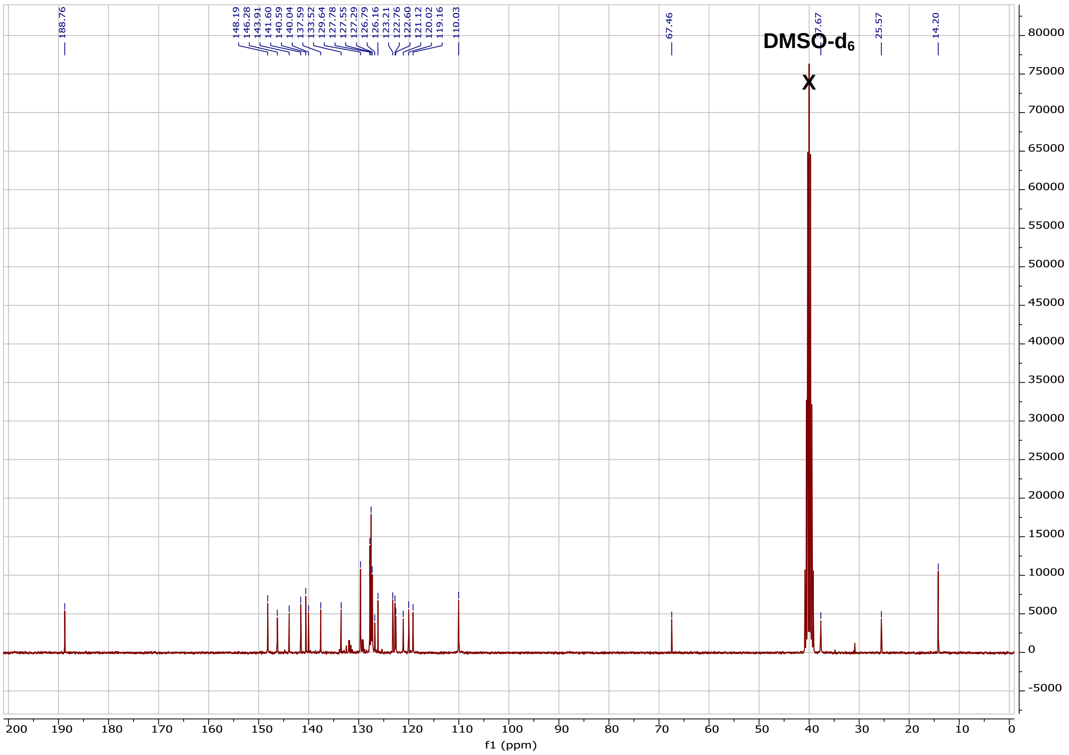

Synthesis of (*E*)-4'-((*E*)-3-(9-ethyl-9H-carbazol-3-yl)acryloyl)-[1,1'-biphenyl]-4-carbaldehyde O-benzoyl oxime **(C2)**

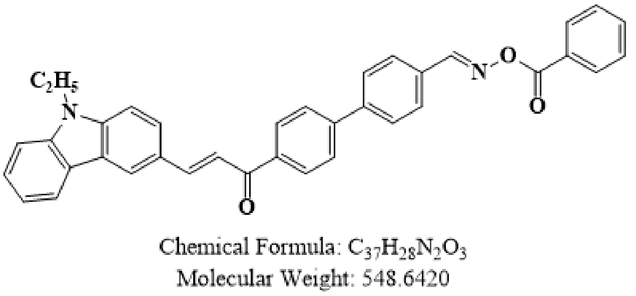

(*E*)-4'-((*E*)-3-(9-ethyl-9H-carbazol-3-yl)acryloyl)-[1,1'-biphenyl]-4-carbaldehyde oxime (1.00 g, 2.61 mmol, M = 382.17 g/mol) was added in anhydrous dichloromethane. Triethylamine (TEA) (1.59 g, 2.19 mL, 15.69 mmol, M = 101.19 g/mol, d = 726 mg/mL) was added to obtain a clear solution. Then, benzoyl chloride (0.23 g, 2.88 mmol, M = 78.50 g/mol, d=1.10 g/mL) was added. The flask was then stirred at room temperature overnight. The solution was subsequently washed with diluted HCl, then water. The organic layer was dried over $MgSO_4$, and the solvent was removed under vacuum. The raw product was recrystallized from dichloromethane/ether to give the product as a solid (0.73 g, 65.8% yield).

^{1}H NMR (300 MHz, $CDCl_3$) δ 8.61 (s, 1H), 8.40 (d, *J* = 1.3 Hz, 1H), 8.20 – 8.13 (m, 5H), 8.13 – 8.07 (m, 1H), 7.92 (t, *J* = 6.7 Hz, 2H), 7.82 (dd, *J* = 8.6, 1.5 Hz, 1H), 7.77 (dd, *J* = 8.3, 7.0 Hz, 4H), 7.66 – 7.60 (m, 2H), 7.54 – 7.48 (m, 3H), 7.43 (d, *J* = 8.7 Hz, 2H), 7.34 – 7.27 (m, 1H), 4.39 (q, *J* = 7.2 Hz, 2H), 1.47 (t, *J* = 7.2 Hz, 3H).

^{13}C NMR (75 MHz, $CDCl_3$) δ 189.92 (s), 163.95 (s), 156.24 (s), 146.73 (s), 143.74 (s), 143.33 (s), 141.56 (s), 140.52 (s), 138.21 (s), 133.48 (s), 129.81 (s), 129.77 (s), 129.17 (s), 129.11 (s), 128.61 (s), 127.76 (s), 127.28 (s), 126.41 (s), 125.92 (s), 123.54 (s), 122.89 (s), 121.78 (s), 120.68 (s), 119.78 (s), 118.90 (s), 108.95 (s), 37.82 (s), 13.86 (s).

HRMS (ESI MS) m/z: theor: 548.6 found: 548.2 ($[M+H]^+$ detected)

^{1}H NMR spectrum of (*E*)-4'-((*E*)-3-(9-ethyl-9H-carbazol-3-yl)acryloyl)-[1,1'-biphenyl]-4-carbaldehyde O-benzoyl oxime **(C2)**

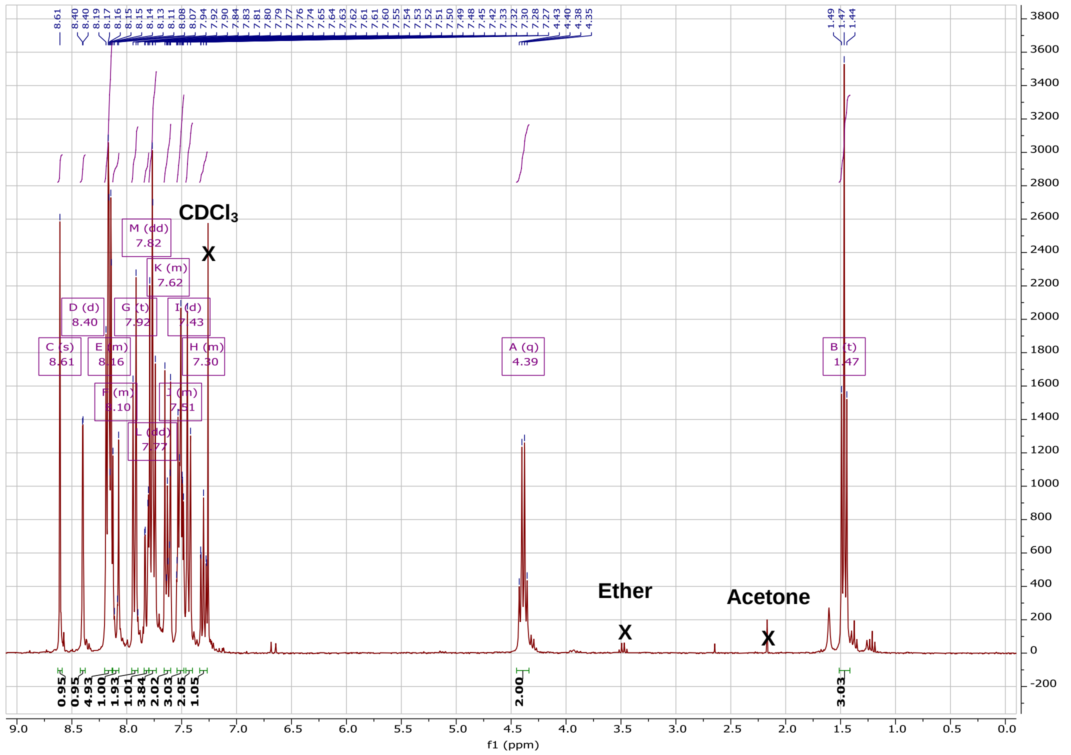

^{13}C NMR spectrum of (*E*)-4'-((*E*)-3-(9-ethyl-9H-carbazol-3-yl)acryloyl)-[1,1'-biphenyl]-4-carbaldehyde O-benzoyl oxime **(C2)**

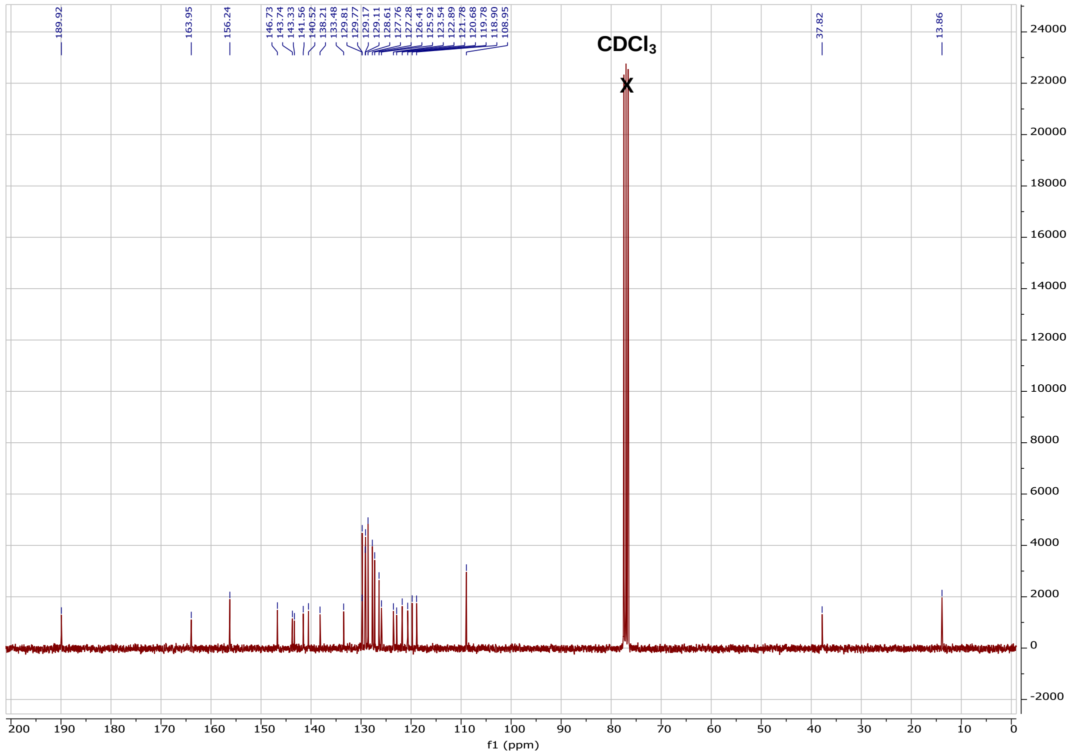

Synthesis of (*E*)-4'-((E)-3-(9-ethyl-9H-carbazol-3-yl)acryloyl)-[1,1'-biphenyl]-4-carbaldehyde O-pivaloyl oxime **(C3)**

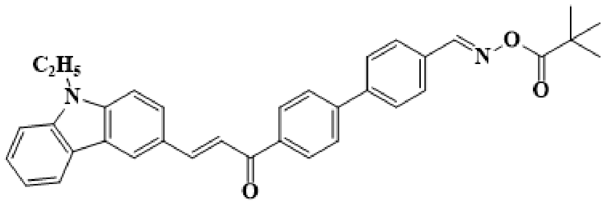

Chemical Formula: $C_{35}H_{32}N_2O_3$
Molecular Weight: 528.6520

(*E*)-4'-((*E*)-3-(9-ethyl-9H-carbazol-3-yl)acryloyl)-[1,1'-biphenyl]-4-carbaldehyde oxime (1.00 g, 2.25 mmol, M = 444.18 g/mol) was added in anhydrous dichloromethane. Triethylamine (1.37 g, 1.88 mL, 13.50 mmol, M = 101.19 g/mol, d = 726 mg/mL) was added to obtain a clear solution. Then, pivaloyl chloride (0.30 g, 0.30 mL, 2.47 mmol, M = 120.58 g/mol, d=0.98 g/m^3) was added. The flask was then stirred at room temperature overnight. The solution was subsequently washed with diluted HCl, then water. The organic layer was dried over $MgSO_4$, and the solvent was removed under vacuum. The raw product was recrystallized from dichloromethane/ether to give the product as a solid (0.97 g, 81.6% yield).

^{1}H NMR (400 MHz, $CDCl_3$) δ 8.44 (s, 1H), 8.39 (d, *J* = 1.5 Hz, 1H), 8.18 – 8.14 (m, 3H), 8.11 – 8.07 (m, 1H), 7.88 – 7.84 (m, 2H), 7.81 (dd, *J* = 8.6, 1.6 Hz, 1H), 7.77 – 7.71 (m, 4H), 7.64 – 7.60 (m, 1H), 7.51 (ddd, *J* = 8.2, 5.3, 1.1 Hz, 1H), 7.43 (dd, *J* = 8.4, 3.5 Hz, 2H), 7.32 – 7.28 (m, 1H), 4.38 (q, *J* = 7.2 Hz, 2H), 1.46 (t, *J* = 7.2 Hz, 3H), 1.36 – 1.33 (s, 9H).

^{13}C NMR (101 MHz, $CDCl_3$) δ 189.92 (s), 175.31 (s), 155.72 (s), 146.71 (s), 143.75 (s), 143.16 (s), 141.56 (s), 140.53 (s), 138.18 (s), 129.94 (s), 129.15 (s), 128.96 (s), 127.69 (s), 127.25 (s), 126.40 (s), 125.94 (s), 123.55 (s), 122.89 (s), 121.75 (s), 120.67 (s), 119.78 (s), 118.93 (s), 108.94 (s), 38.46 (s), 37.81 (s), 27.24 (s), 13.84 (s).

HRMS (ESI MS) m/z: theor: 528.7 found: 528.2 ([M+H]$^+$ detected)

^{1}H NMR spectrum of (*E*)-4'-((*E*)-3-(9-ethyl-9H-carbazol-3-yl)acryloyl)-[1,1'-biphenyl]-4-carbaldehyde O-pivaloyl oxime **(C3)**

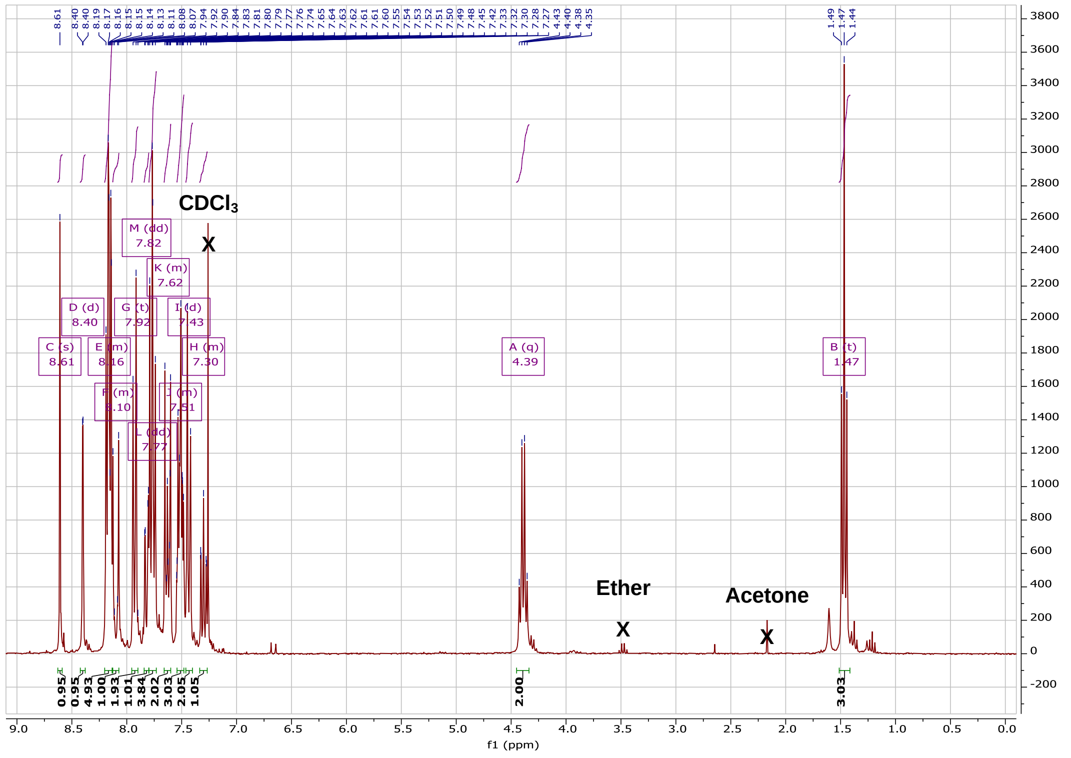

^{13}C NMR spectrum of (*E*)-4'-((*E*)-3-(9-ethyl-9H-carbazol-3-yl)acryloyl)-[1,1'-biphenyl]-4-carbaldehyde O-pivaloyl oxime **(C3)**

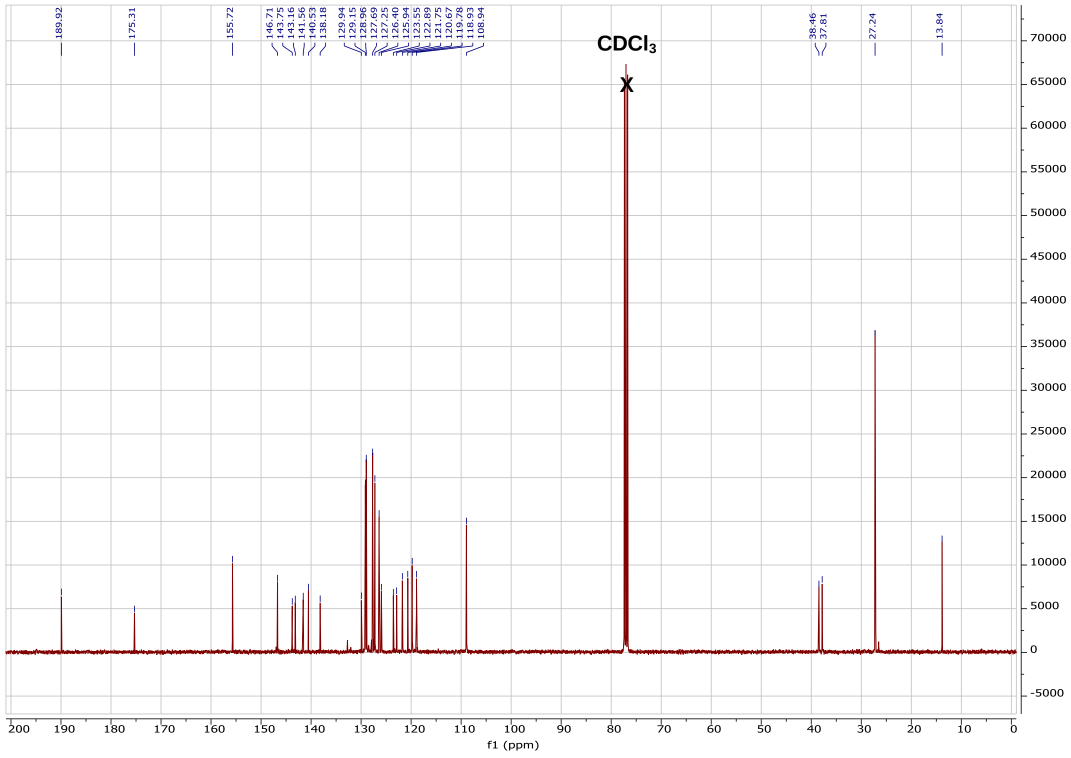

Synthesis of ethyl 2-(6-((E)-3-(4'-((E)-((benzoyloxy)imino)methyl)-[1,1'-biphenyl]-4-yl)-3-oxoprop-1-en-1-yl)-9-ethyl-9H-carbazol-3-yl)-2-oxoacetate **(C4)**

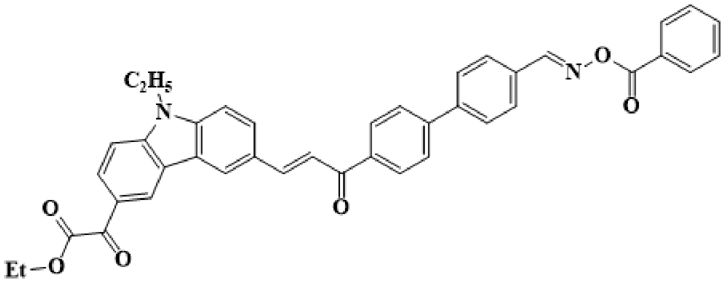

Chemical Formula: $C_{41}H_{32}N_2O_6$
Molecular Weight: 648.7150

(*E*)-4'-((*E*)-3-(9-ethyl-9H-carbazol-3-yl)acryloyl)-[1,1'-biphenyl]-4-carbaldehyde O-benzoyl oxime (0.5 g, 0.95 mmol, M = 528.65 g/mol) was dissolved in anhydrous dichloromethane (100 mL). Then, aluminum trichloride ($AlCl_3$) (1.26 g, 9.46 mmol, M = 133.33 g/mol) and ethyl chlorooxoacetate (1.06 mL, 9.46 mmol, M = 136.53 g/mol, d = 1.22 g/mL) were added successively into the mixture at 0-5 ^{o}C. After stirring for overnight at room temperature, the solution was subsequently washed with water and dried over $MgSO_4$. After evaporation of the volatiles, the raw product was recrystallized from dichloromethane/ether to give the product as a solid (0.52 g, 87.5% yield).

^{1}H NMR (400 MHz, $CDCl_3$) δ 8.84 (d, *J* = 1.5 Hz, 1H), 8.60 (s, 1H), 8.44 (s, 1H), 8.20 – 8.17 (m, 2H), 8.16 – 8.13 (m, 2H), 8.06 (d, *J* = 15.6 Hz, 1H), 7.92 (d, *J* = 8.3 Hz, 2H), 7.83 (dd, *J* = 5.4, 3.3 Hz, 1H), 7.76 (dd, *J* = 13.1, 8.4 Hz, 4H), 7.65 (dd, *J* = 11.1, 8.4 Hz, 2H), 7.61 (dd, *J* = 4.9, 3.7 Hz, 1H), 7.52 – 7.45 (m, 4H), 4.53 (q, *J* = 7.1 Hz, 2H), 4.44 – 4.38 (m, 2H), 1.48 (t, *J* = 7.2 Hz, 6H).

^{13}C NMR (101 MHz, $CDCl_3$) δ 189.59 (s), 185.57 (s), 164.53 (s), 163.91 (s), 156.18 (s), 145.65 (s), 144.22 (s), 143.91 (s), 143.18 (s), 142.18 (s), 137.83 (s), 133.45 (s), 129.72 (s), 129.18 (s), 129.07 (s), 128.57 (s), 128.41 (s), 127.79 (s), 127.71 (s), 127.58 (s), 127.29 (s), 124.54 (s), 124.29 (s), 123.70 (s), 122.93 (s), 121.39 (s), 119.90 (s), 109.68 (s), 109.10 (s), 62.25 (s), 38.23 (s), 14.18 (s), 13.84 (s).

HRMS (ESI MS) m/z: theor: 648.7 found: 648.3 ([M+H]$^+$ detected)

^{1}H NMR spectrum of (*E*)-4'-((*E*)-3-(9-ethyl-6-(2-oxobutanoyl)-9H-carbazol-3-yl)acryloyl)-[1,1'-biphenyl]-4-carbaldehyde O-benzoyl oxime **(C4)**

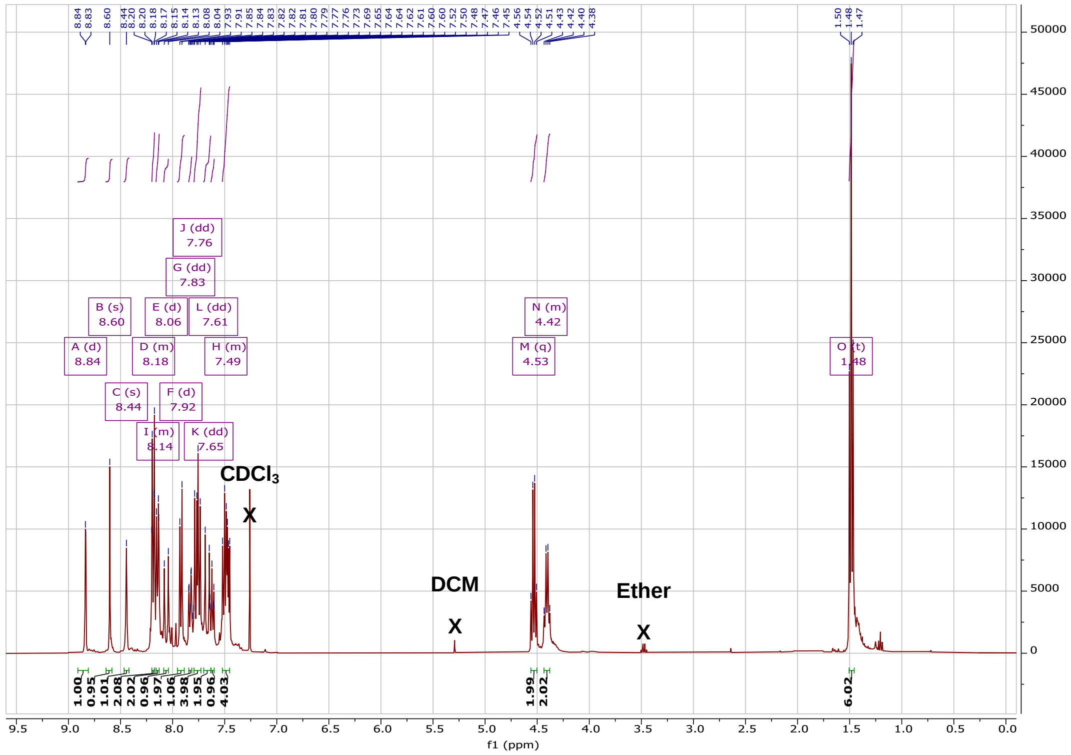

^{13}C NMR spectrum of (*E*)-4'-((*E*)-3-(9-ethyl-6-(2-oxobutanoyl)-9H-carbazol-3-yl)acryloyl)-[1,1'-biphenyl]-4-carbaldehyde O-benzoyl oxime **(C4)**

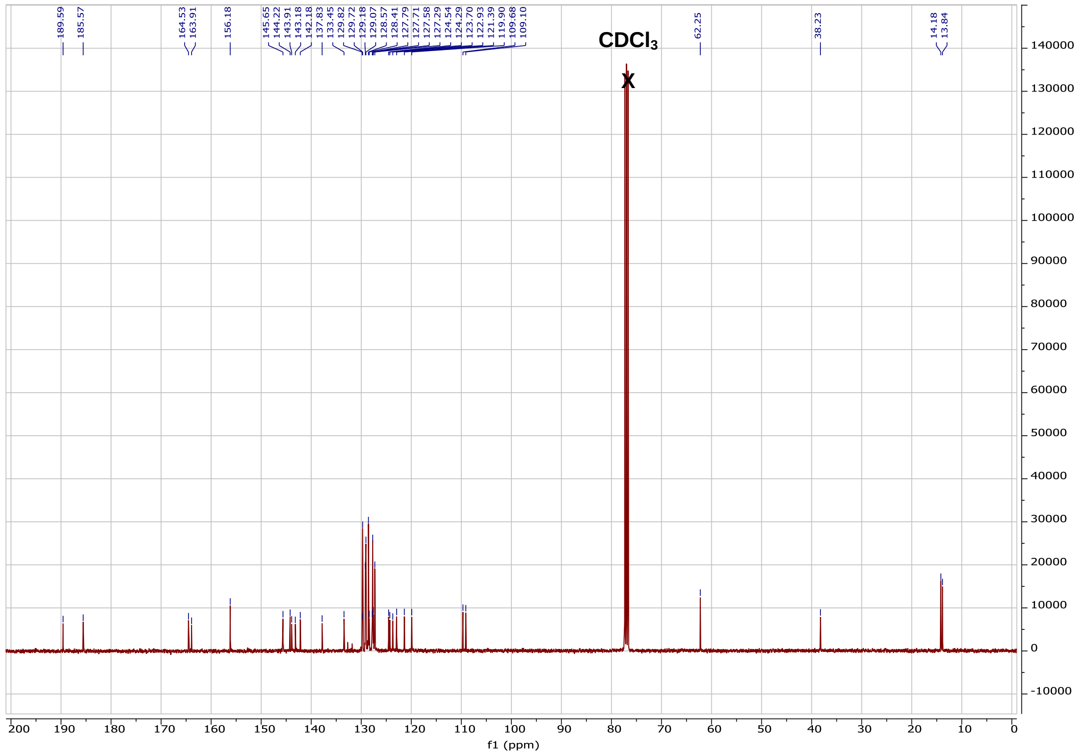

Synthesis of ethyl 2-(9-ethyl-6-((*E*)-3-oxo-3-(4'-((*E*)-((pivaloyloxy)imino)methyl)-[1,1'-biphenyl]-4-yl)prop-1-en-1-yl)-9H-carbazol-3-yl)-2-oxoacetate **(C5)**

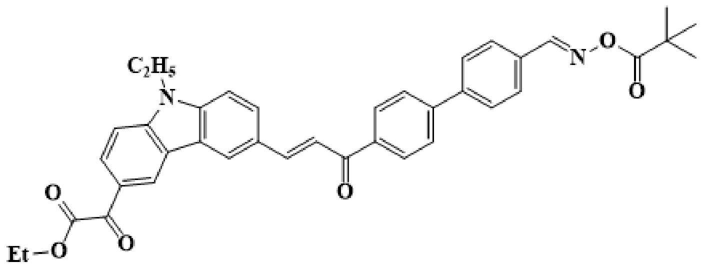

Chemical Formula: $C_{39}H_{36}N_2O_6$
Molecular Weight: 628.7250

(*E*)-4'-((*E*)-3-(9-ethyl-9H-carbazol-3-yl)acryloyl)-[1,1'-biphenyl]-4-carbaldehyde O-pivaloyl oxime (0.5 g, 0.91 mmol, M = 548.64 g/mol) was dissolved in anhydrous dichloromethane (100 mL). Then, aluminum trichloride (1.22 g, 9.11 mmol, M = 133.33 g/mol) and ethyl chlorooxoacetate (1.02 mL, 9.11 mmol, M = 136.53 g/mol, d = 1.22 g/mL) were added successively into the mixture at 0-5 ^{o}C. After stirring for overnight at room temperature, the solution was subsequently washed with water and dried over $MgSO_4$. After evaporation of the volatiles, the raw product was recrystallized from dichloromethane/ether to give the product as a solid (0.53 g, 89.7% yield).

^{1}H NMR (400 MHz, $CDCl_3$) δ 8.82 (d, *J* = 1.5 Hz, 1H), 8.43 (d, *J* = 6.6 Hz, 2H), 8.17 (d, *J* = 7.8 Hz, 3H), 8.05 (d, *J* = 15.5 Hz, 1H), 7.85 (s, 1H), 7.82 (d, *J* = 7.8 Hz, 1H), 7.74 (dd, *J* = 18.1, 8.2 Hz, 4H), 7.69 – 7.63 (m, 2H), 7.46 (dd, *J* = 8.5, 4.9 Hz, 2H), 4.55 – 4.50 (m, 2H), 4.39 (d, *J* = 7.1 Hz, 2H), 1.48 (t, *J* = 7.1 Hz, 6H), 1.34 (s, 9H).

^{13}C NMR (101 MHz, $CDCl_3$) δ 189.62 (s), 185.56 (s), 175.34 (s), 164.52 (s), 155.68 (s), 145.66 (s), 144.20 (s), 143.92 (s), 142.99 (s), 142.16 (s), 137.76 (s), 132.69 (s), 129.93 (s), 129.27 (s), 129.16 (s), 128.93 (s), 127.64 (s), 127.56 (s), 127.25 (s), 124.51 (s), 124.25 (s), 123.67 (s), 122.91 (s), 121.37 (s), 119.88 (s), 109.67 (s), 109.09 (s), 65.80 (s), 62.24 (s), 38.42 (s), 38.22 (s), 27.18 (s), 27.02 (s), 15.20 (s), 14.16 (s), 13.83 (s).

HRMS (ESI MS) m/z: theor: 628.7 found: 628.3 ($[M+H]^+$ detected)

^{1}H NMR spectrum of (*E*)-4'-((*E*)-3-(9-ethyl-6-(2-oxobutanoyl)-9H-carbazol-3-yl)acryloyl)-[1,1'-biphenyl]-4-carbaldehyde O-pivaloyl oxime **(C5)**

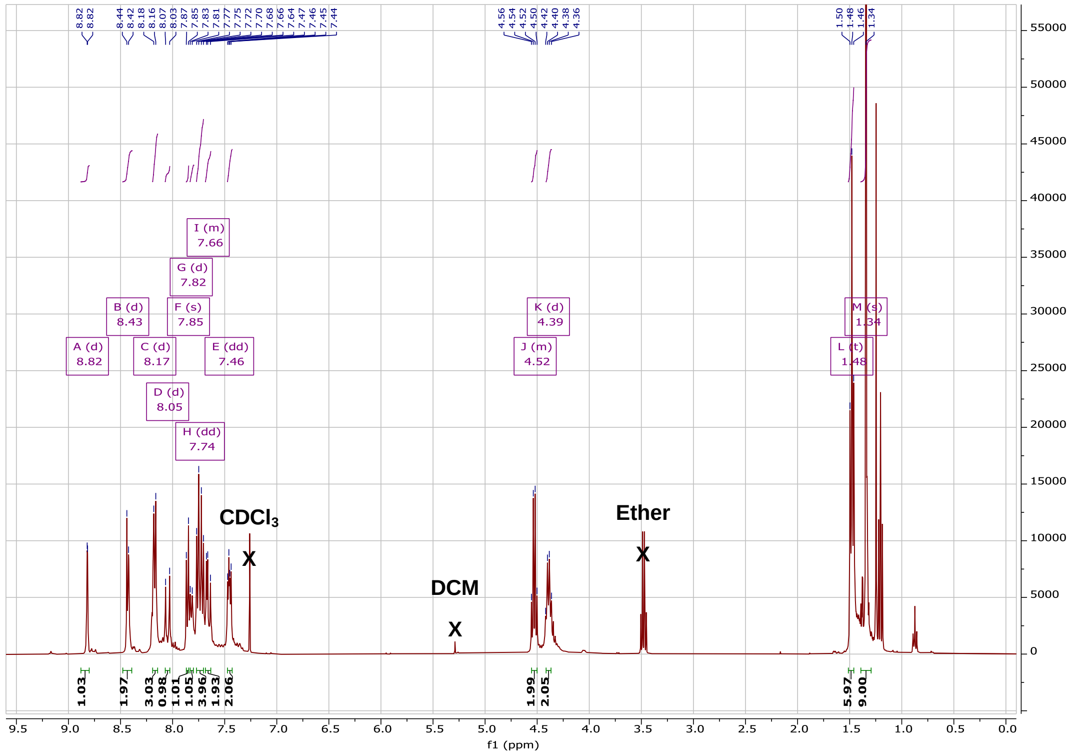

^{13}C NMR spectrum of (*E*)-4'-((*E*)-3-(9-ethyl-6-(2-oxobutanoyl)-9H-carbazol-3-yl)acryloyl)-[1,1'-biphenyl]-4-carbaldehyde O-pivaloyl oxime **(C5)**

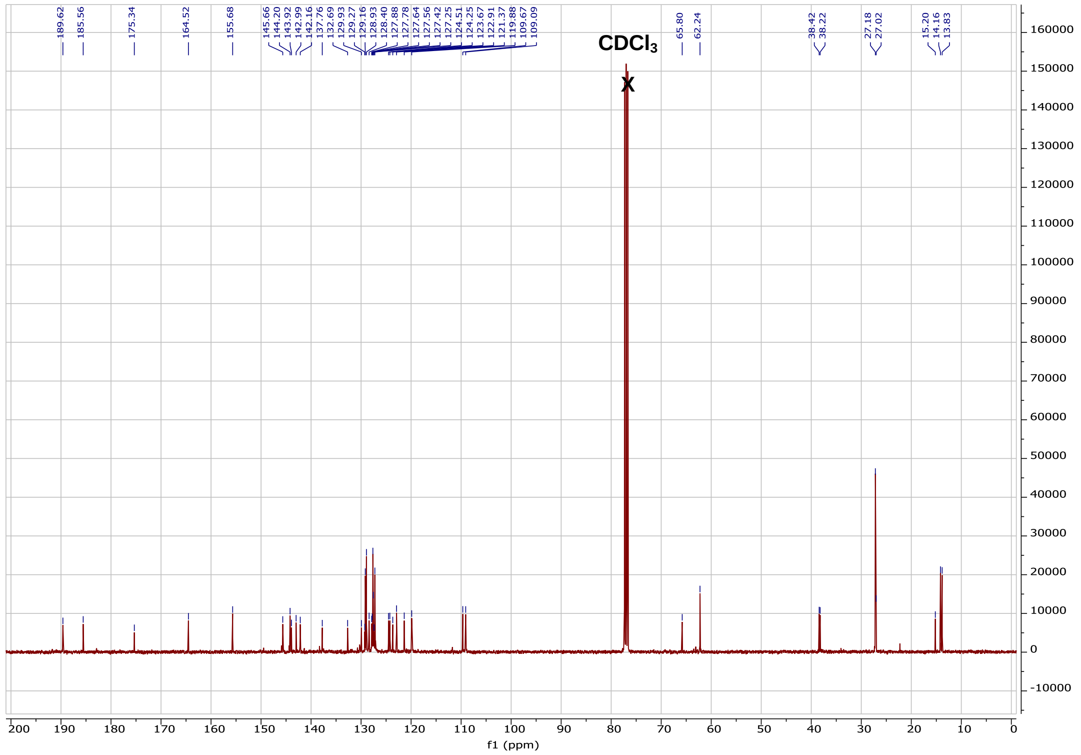

Supplement: Supplementary file 1 — Supporting Information [file ANIE-64-e202425598-s001.pdf]
